# Impact of Carbon Fixation, Distribution and Storage on the Production of Farnesene and Limonene in *Synechocystis* PCC 6803 and *Synechococcus* PCC 7002

**DOI:** 10.3390/ijms25073827

**Published:** 2024-03-29

**Authors:** Marine Vincent, Victoire Blanc-Garin, Célia Chenebault, Mattia Cirimele, Sandrine Farci, Luis Fernando Garcia-Alles, Corinne Cassier-Chauvat, Franck Chauvat

**Affiliations:** 1 Institute for Integrative Biology of the Cell (I2BC), Université Paris-Saclay, CEA, CNRS, 91198 Gif-sur-Yvette, France; marinevt@orange.fr (M.V.); victoire.blancgarin@gmail.com (V.B.-G.); celia.chenebault@gmail.com (C.C.); mattia.cirimele@ens-paris-saclay.fr (M.C.); sandrine.farci@cea.fr (S.F.); corinne.cassier-chauvat@cea.fr (C.C.-C.); 2 Université Paris-Saclay, ENS Paris-Saclay, 91190 Gif-sur-Yvette, France; 3 Toulouse Biotechnology Institute, Université de Toulouse, CNRS, INRAE, INSA, 135 Avenue de Rangueil, 31077 Toulouse, France; lgarciaa@insa-toulouse.fr

**Keywords:** *Synechocystis* PCC 6803, *Synechococcus* PCC 7002, genome editing, farnesene, limonene, RubisCO, phosphoribulokinase, carboxysome, geranylgeranyl pyrophosphate synthase, β-carotene hydroxylase, γ-carotene hydroxylase

## Abstract

Terpenes are high-value chemicals which can be produced by engineered cyanobacteria from sustainable resources, solar energy, water and CO_2_. We previously reported that the euryhaline unicellular cyanobacteria *Synechocystis* sp. PCC 6803 (S.6803) and *Synechococcus* sp. PCC 7002 (S.7002) produce farnesene and limonene, respectively, more efficiently than other terpenes. In the present study, we attempted to enhance farnesene production in S.6803 and limonene production in S.7002. Practically, we tested the influence of key cyanobacterial enzymes acting in carbon fixation (RubisCO, PRK, CcmK3 and CcmK4), utilization (CrtE, CrtR and CruF) and storage (PhaA and PhaB) on terpene production in S.6803, and we compared some of the findings with the data obtained in S.7002. We report that the overproduction of RubisCO from S.7002 and PRK from *Cyanothece* sp. PCC 7425 increased farnesene production in S.6803, but not limonene production in S.7002. The overexpression of the *crtE* genes (synthesis of terpene precursors) from S.6803 or S.7002 did not increase farnesene production in S.6803. In contrast, the overexpression of the *crtE* gene from S.6803, but not S.7002, increased farnesene production in S.7002, emphasizing the physiological difference between these two model cyanobacteria. Furthermore, the deletion of the *crtR* and *cruF* genes (carotenoid synthesis) and *phaAB* genes (carbon storage) did not increase the production of farnesene in S.6803. Finally, as a containment strategy of genetically modified strains of S.6803, we report that the deletion of the *ccmK3K4* genes (carboxysome for CO_2_ fixation) did not affect the production of limonene, but decreased the production of farnesene in S.6803.

## 1. Introduction

**Figure 1:**
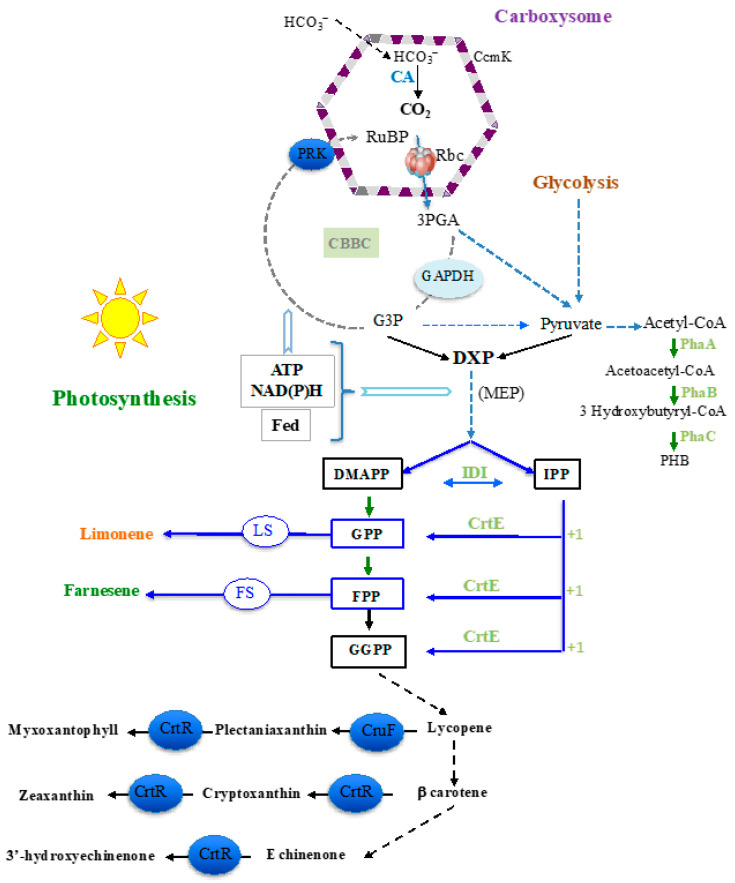
Schematic representation of the metabolic pathway and key compounds involved in the synthesis of terpenes from CO_2_. Abbreviations: CA carbonic anhydrase; CBBC: Calvin–Benson–Bassham cycle (consuming 1 NADPH, 1 ATP); CrtE: geranylgeranyl pyrophosphate synthase; DMAPP: dimethylallyl pyrophosphate; DXP: 1-deoxy-D-xylulose-5-phosphate; Fed: ferredoxin; FPP: farnesyl pyrophosphate; FS: farnesene synthase; GAPDH: glyceraldehyde-3P-dehydrogenase; GPP: geranyl pyrophosphate; GPP: geranyl pyrophosphate; GGPP: geranylgeranyl pyrophosphate; G3P: glyceraldehyde-3-phosphate; 3PGA: 3-phosphoglycerate; IPP: isopentenyl pyrophosphate; IDI: isopentenyl-diphosphate isomerase; LS: limonene synthase; MEP: methylerythritol 4-phosphate (it consumes 2 NADPH, 1 ATP and 1 CTP); phaA: acetyl-CoA acetyltransferase; phaB: Acetoacetyl-CoA reductase; phaC: PHB synthase; PRK: phosphoribulokinase; Rbc: ribulose biphosphate carboxylase (RubisCO); RuBP: ribulose-1,5-bisphosphate. The genes manipulated in this study encode the CrtE, CrtR, CruF, PhaA, PhaB, PRK and RubisCO enzymes, as well as the CcmK3 and CcmK4 carboxysome shell proteins.

Terpenes constitute a large family of chemicals often produced by plants (attraction of pollinators and/or protection against pathogens) in low quantities and at high prices. They are used for the production of fragrances, pharmaceuticals, pesticides, solvents and potentially biofuels [1,2,3]. All terpenes (general formula (C_5_H_8_)_n_) derive from the five-carbon (C5) building blocks dimethylallyl pyrophosphate (DMAPP) and isopentenyl pyrophosphate (IPP). The head-to-tail covalent linkage of DMAPP and IPP catalyzed by the geranyl diphosphate synthase (GPPS) enzyme generates the geranyl diphosphate (GPP) precursor of monoterpenes (C_10_H_16_), such as limonene. Then, the addition of another IPP unit on GPP catalyzed by the farnesyl diphosphate synthase (FPPS) forms the farnesyl pyrophosphate (FPP) precursor of sesquiterpenes (C_15_H_24_), such as farnesene (Figure 1).

In addition to plants, which are better used for food, cyanobacteria, the robust photosynthetic prokaryotes colonizing our planet, can be used for the ecologically responsible production of terpenes from sunlight and CO_2_ (for reviews see [1,2,3]). Cyanobacteria fix CO_2_ using the Calvin–Benson–Bassham (CBB) pathway in which the key enzyme ribulose bisphosphate carboxylase/oxygenase (RubisCO) catalyzes the addition of one molecule of CO_2_ to one molecule of ribulose biphosphate produced by the other key enzyme phosphoribulokinase (PRK). In parallel, they have developed the carboxysome subcellular compartment (assembled from various shell proteins such as CcmK3 and CcmK4) to encapsulate RubisCO in a CO_2_-rich environment that favors its carbon-fixing (carboxylase) activity over its detrimental oxygenase activity (for reviews see [4,5]). A part of the photosynthetically fixed carbon is used by the methylerythritol-4-phosphate (MEP) pathway to produce DMAPP and IPP (Figure 1), which are transformed by a geranylgeranyl pyrophosphate synthase (CrtE) into GPP, FPP and GGPP, which is used by the carotene hydroxylases (CrtR and CruF) to produce chlorophyll and carotenoids [2,3,6]. GPP and FPP can also be transformed into terpenes, following introduction and expression in cyanobacteria of synthetic terpene-synthase encoding genes adapted to the cyanobacterial codon usage [2,3]. The unicellular euryhaline cyanobacteria *Synechocystis* sp. PCC 6803 (hereafter S.6803) and *Synechococcus* sp. PCC 7002 (S.7002, coastal organism) are interesting for this purpose. They have good genetics (one can easily delete or overexpress genes of interests) and they grow well in sea and brackish waters to preserve freshwater resources for agriculture [7].

In the frame of our exploration of the ability of physiologically different cyanobacteria for the photoproduction of chemically different terpenes, we previously codon-adapted several genes encoding terpene synthases, in an RSF1010-derived replicative plasmid or in a neutral chromosome site [8,9,10,11]. The results showed that S.6803 produces farnesene better than bisabolene, limonene, santalene and pinene, whereas S.7002 produces limonene more efficiently than bisabolene and pinene [9]. In the present study, we tested and compared several genetic strategies to address the improvement of farnesene production in S.6803 and limonene production in S.7002 [11].

## 2. Results and Discussion

### 2.1. The Overexpression of the RubisCO Genes from Synechococcus PCC 7002 Increases Farnesene Production in Synechocystis PCC 6803

Previous studies showed that overexpressing the RubisCO-encoding genes can improve cell growth and/or the photosynthetic production of high value chemicals in S.6803 and/or S.7002 [12,13,14,15]. In this study, we attempted to improve farnesene production in S.6803 by introducing an extra copy of the RubisCO-encoding genes in the S.6803-ChrFS expressing the *Picea abies* α-farnesene synthase gene from a neutral site of its chromosome [9]. To increase the odds of improving RubisCO activity in S.6803, we used extra RubisCO genes from not only S.6803, but also physiologically diverse cyanobacteria studied in our laboratory. These are *Cyanothece* sp. PCC 7425 (hereafter C.7425) and S.7002 where their RubisCO genes constitute the *rbcLXS* operon encoding the large (RbcL) and small (RbcS) RubisCO subunits, and the RbcX chaperon (See Cyanobase). We also used the *rbcLS* operon of *Synechococcus elongatus* PCC 7942 (S.7942) where *rbcX* is located away from *rbcLS* [16,17].

**Figure 2:**
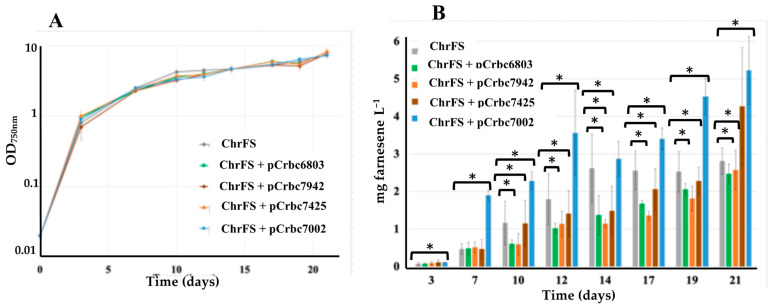
Simultaneous analysis of growth (**A**) and farnesene production (**B**) of the *Synechocystis* PCC 6803 strains harboring the strongly expressed genes encoding farnesene-synthase (FS) in a neutral chromosomal site (chrFS) and the RubisCO (*rbc*) genes, in a pC-derived replicative plasmid (pCrbc). These plasmids encode the RubisCO enzymes from the distantly related unicellular cyanobacteria *Cyanothece* PCC 7425 (pCrbc7425), *Synechococcus* PCC 7002 (pCrbc7002), *Synechococcus* PCC 7942 (pCrbc7942) and *Synechocystis* PCC 6803 (pCrbc6803). Cells were grown under standard photoautotrophic conditions in the presence of a dodecane overlay (20% *v*/*v*) to assay terpene production for 21 days. Error bars represent standard deviation from biological triplicates. They are too small to be visible in panel (**A**). The hooks ⊏ indicate a significant difference between the two compared experiments (*t*-test, *p* < 0.05, symbolized by *).

These RubisCO operons were PCR amplified from their respective cyanobacterial genomes (Figure S1) using oligonucleotides (Table S1) that introduced convenient restriction site upstream of the ATG start codon of *rbcL* (*Nde*I for S.6803, S.7002 and S.7942, and XhoI for C.7425) and downstream of the TAA stop codon of *rbcS* (*EcoR*I for S.6803, *Pvu*II for both S.7002 and S.7942, and *BspE*I for C.7425). After restriction, the RubisCO operons were cloned downstream of the strong p*R* promoter of the RSF1010-derived replicative pC plasmid vector [9] opened with the same enzymes. The resulting Sm^R^/Sp^R^ plasmids, pCrbc6803, pCrbc7002, pCrbc7942 and pCrb7425 (Figure S1 and Table S2) and the (empty) pC control vector were introduced by conjugation in the S.6803-ChrFS engineered strain that produces farnesene [9]. In each case, two independent Sm^R^/Sp^R^ clones were selected and analyzed by PCR and DNA sequencing (Figure S1). The data showed that all these pC-derived plasmids, pCrbC7425, pCrbc7942, pCrbc6803 and pCrbc7002, replicate stably in S.6803 with no impact on cell growth (Figure 2), similar to the pC control vector 7002 [11]. The resulting recombinant strains, which also produce RubisCO from their indigenous chromosomal RubisCO genes, are represented in Figure S2.

The production of farnesene by the S.6803-chrFS strains propagating the RubisCO-producing plasmids pCrbc6803, pCrbc7002, pCrbc7425 and pCrbc7942 was measured over 21-day periods of photoautotrophic growth, as previously described [9]. GC–MS analysis of the terpene-trapping dodecane overlay samples from all tested strains showed a peak with similar retention time and ion chromatogram as a pure standard of α-farnesene. As compared to the S.6803-chrFS control strain, which has a natural level of RubisCO, the level of farnesene production driven by the pCrbc7002 plasmid showed that high-level expression of the RubisCO genes from S.7002 enhanced farnesene production (about 2-fold at day 21, Figure 2). Unlike pCrbc7002, the pCrbc6803, pCrbc7425 and pCrbc7942 plasmids did not increase farnesene production, though they express their RubisCO genes from the same strong promoter and ribosome binding site as pCrbc7002. Together, these findings suggest that the RubisCO of S.7002 is more active in S.6803 than the other tested RubisCO, including the endogenous enzyme of S.6803, which cannot be degraded by a possible protease recognizing foreign proteins.

### 2.2. The Overexpression of the Phosphoribulokinase Gene from Cyanothece PCC 7425 Increases Farnesene Production in Synechocystis PCC 6803

As the elimination of the CP12 negative regulator of the phosphoribulokinase (PRK) enzyme was shown to improve terpene production in S.6803 [10], we decided to overexpress the *prk* gene as an attempt to enhance farnesene production. To increase the odds of enhancing PRK activity, we decided to clone in the pC plasmid vector an extra copy of *prk* from not only S.6803, but also C.7425, S.7002 and S.7942. These *prk* genes were PCR amplified from their respective cyanobacterial genome using oligonucleotide primers (Table S1) that embedded their ATG start codon in a *Nde*I restriction site (CATATG), and introduced an *Eco*RI site downstream of their stop codons: TAA (S.6803) or TAG (S.7002, S.7942 and C.7425). After restriction with both *Nde*I and *Eco*RI, these genes were cloned in pC opened with the same enzymes, and transformed to *E. coli*. All attempts to clone the *prk* genes from S.6803, S.7002 and S.7942 in pC were unsuccessful, indicating that cyanobacterial PRKs are toxic to *E. coli*. Supporting this interpretation, previous cloning in *E.coli* of the *prk* gene from S.7942 [18] and S.6803 [19], for in vitro analysis of PRK, employed an inducible promoter system that allowed for the decoupling of *E.coli* growth from the production of PRK.

**Figure 3:**
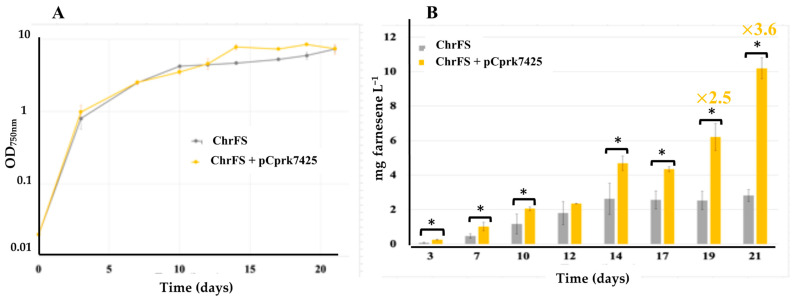
Simultaneous analysis of growth (**A**) and farnesene production (**B**) of the *Synechocystis* PCC 6803 strain harboring the strongly expressed genes encoding farnesene synthase (FS) and phoshoribulokinase (prk) in a neutral chromosomal site (chrFS) and a pC-derived replicative plasmid, respectively. This pCprk7425 plasmid encodes the PRK enzyme from *Cyanothece* PCC 7425, a unicellular cyanobacterium distantly related to both *Synechocystis* PCC 6803 and *Synechococcus* PCC 7002. Cells were grown under standard photoautotrophic conditions in the presence of a dodecane overlay (20% *v*/*v*) to assay terpene production for 21 days. Error bars represent standard deviation from biological triplicates. They are too small to be visible in panel (**A**). The hooks ⊏ indicate a significant difference between the two compared experiments (*t*-test, *p* < 0.05, symbolized by *).

Nevertheless, we were able to clone the *prk* gene from C.7425 (prk7425) downstream of the strong constitutive p*R* promoter of the pC plasmid (Table S2), suggesting that PRK_7425_ is somehow less active in *E. coli* than the other cyanobacterial PRKs presently tested. The resulting plasmid, pCprk7425 (Table S2), was introduced by conjugation in the S.6803-chrFS strain expressing the farnesene synthase gene from its chromosome [9]. A Sm^R^/Sp^R^ clone (Figure S2) was analyzed. It showed that pCprk7425 does not alter the growth of S.6803 (Figure 3). The production of farnesene during the photoautotrophic growth of the S.6803-chrFS-pCprk7425 reporter strain (Figure S2) was analyzed for 21 days and compared to that of the S.6803-chrFS control strain. The data showed that increasing the expression of *prk* enhances (about three-fold) the production of farnesene in S.6803 (Figure 3). This result is consistent with both our previous report on the CP12-deleted mutant [10] and the above finding that overexpressing RubisCO genes improved farnesene production in S.6803 (Figure 2). Collectively, these data indicate that increasing CO_2_ fixation enhances terpene production in S.6803.

### 2.3. The Overexpression of the Genes Encoding RubisCO and PRK Enzymes Does Not Increase Limonene Production in Synechococcus PCC 7002

**Figure 4:**
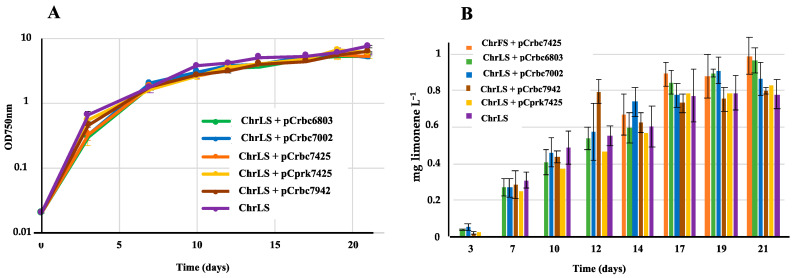
Simultaneous analysis of growth (**A**) and limonene production (**B**) of the *Synechococcus* PCC 7002 strains harboring the strongly expressed limonene-synthase gene (LS) in a neutral chromosomal site (chrLS) and a pC-derived plasmid expressing either the phoshoribulokinase gene from *Cyanothece* PCC 7425 (pCprk7425) or the RubisCO genes from *Cyanothece* PCC 7425 (pCrbc7425), *Synechococcus* PCC 7002 (pCrbc7002), *Synechococcus* PCC 7942 (pCrbc7942) or *Synechocystis* PCC 6803 (pCrbc6803). Cells were grown under standard photoautotrophic conditions in the presence of a dodecane overlay (20% *v*/*v*) to assay terpene production for 21 days. Error bars represent standard deviation from biological triplicates. They are too small to be visible in panel A.

We also attempted to improve limonene production in the previously described S.7002-chrLS strain that carries the *Mentha spicata* 4S-limonene synthase gene in a neutral chromosome site [11]. Therefore, the above-described plasmids overexpressing the studied phosphoribulokinase gene (pCprk7425) or RubisCO operons (pCrbc6803, pCrbc7002, pCrbC7425 and pCrbc7942) were introduced by conjugation in S.7002-chrLS. The growth and limonene production of the resulting reporter strains (Figure S3) appeared to be similar to that of the S.7002-chrLS control strain (Figure 4).

Collectively, the present findings show that the photoautotrophic growth of both S.6803 and S.7002 is not limited by the natural abundance of their indigenous enzymes RubisCO and PRK. In contrast, the production of terpenes is limited by the natural abundance of both RubisCO and PRK in S.6803 (Figure 2 and Figure 3), but not in S.7002 (Figure 4). These differences remind us that a model cyanobacterium is essentially a model of itself. Consequently, it is therefore important to study several cyanobacteria in parallel (in the same laboratory) to better understand their common and specific properties, and exploit the resulting knowledge for biotechnological purposes.

### 2.4. The Overexpression of the crtE Genes from Synechocystis PCC 6803 or Synechococcus PCC 7002 Decreases the Production of Both Farnesene and Limonene in Synechocystis PCC 6803

**Figure 5:**
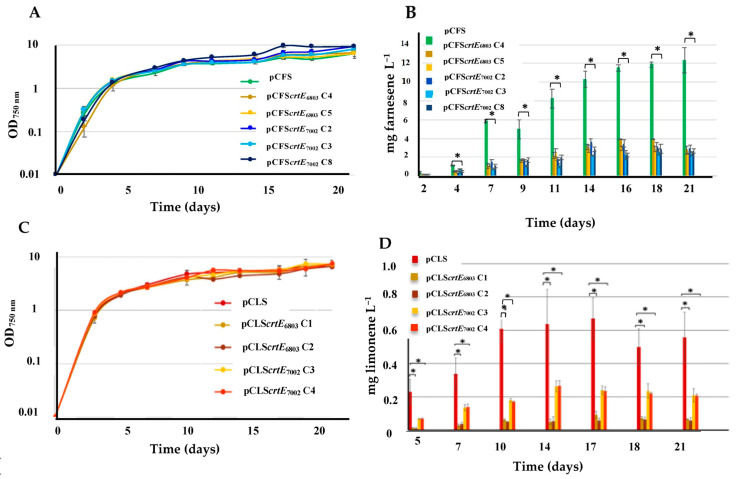
Simultaneous analysis of growth (**A**,**C**) and terpene production (**B**,**D**) of *Synechocystis* PCC 6803 strains harboring a pC-derived plasmid strongly expressing either the farnesene synthase gene (pCFS; (**A**,**B**)) alone or transcriptionally fused to the *crtE* gene from either *Synechocystis* PCC 6803 (pCFS*crtE*_6803_) or *Synechococcus* PCC 7002 (pCFS*crtE*_7002_), or the limonene synthase gene (pCLS; (**C**,**D**)) alone or transcriptionally fused to a *crtE* gene (pCLS*crtE*_6803_ or pCLS*crtE*_7002_). Several clones were studied, as indicated by C1-C8. Cells were grown under standard photoautotrophic conditions in the presence of a dodecane overlay (20% *v*/*v*) to assay terpene production for 21 days. Error bars represent standard deviation from biological triplicates. They are too small to be visible in panels (**A**,**C**). The hooks ⊏ indicate a significant difference between the two compared experiments (*t*-test, *p* < 0.05, symbolized by *).

One of the challenges in using cyanobacteria for terpene production is the competition for prenyl pyrophosphates (GPP, FPP and GGPP) between the synthesis of photosynthetic pigments and the intended terp [20] ene (Figure 1). As most cyanobacteria, such as S.6803 and S.7002, use a single GGPP synthase (named CrtE) for the sequential synthesis of GPP, FPP and GGPP [6,21,22,23], several authors overexpressed synthetic genes encoding FPPS (IspA) from *E. coli* [24,25,26] or GPPS from plants [27,28,29,30] to improve (2–3 fold) terpene production in S.6803, S.7942 (S.7002 was not tested) or S*ynechococcus elongatus* UTEX 2973. These moderate improvements could result from these heterologous enzymes having a low activity and/or stability in cyanobacteria. This eventuality prompted us to test the influence on terpene production of the CrtE enzymes from both S.6803 and S.7002, which have structural differences [6,23]. Therefore, the protein coding sequences of the *crtE*_S.6803_ and *crtE*_S.7002_ genes were PCR amplified from the genomes of S.6803 or S.7002 using specific oligonucleotides (Table S1) which flanked them with a unique restriction site upstream of their start codon, and another unique restriction site behind their stop codon (Table S2). After restriction, these DNA fragments were cloned downstream the *Picea abies* α-farnesene synthase gene (FS) or *Mentha spicata* 4S-limonene synthase gene (LS) of our previously described pCFS and pCLS plasmids [8,9] opened with the same enzymes (*Xho*I and *EcoR*I for pCFS, and *EcoR*I and *Ac*lI for pCLS). The resulting plasmids (Table S2) pCFS*crtE*_6803_ and pCFS*crtE*_7002_ (expression of the FS*crtE*6803 and FS*crtE*7002 operons, respectively), and pCLS*crtE*_6803_ and pCLS*crtE*_7002_ (expression of the LS*crtE*6803 or LS*crtE*7002 operons) were introduced by conjugation in S.6803 (Figure S4), where they appeared to replicate stably (Figure S5). The resulting S.6803 reporter strains (Figure S4), which also expressed their indigenous *crtE* gene from their chromosome, grew similarly to the control strains propagating the pCFS or pCLS control plasmids (Figure 5).

The production of farnesene directed by the pCFS*crtE*_6803_ and pCFS*crtE*_7002_ plasmids was lower (about three-fold) than the level driven by the pCFS control plasmid (Figure 5). Similarly, the production of limonene directed by the pCLS*crtE*_6803_ and pCLS*crtE*_7002_ plasmids were smaller (about six-fold and three-fold, respectively) than the level driven by the pCLS control plasmid (Figure 5). Collectively, these data indicate that the natural abundance of the CrtE enzyme is not limiting terpene production in S.6803. The present finding that an increased level of CrtE actually decreases terpene production suggests that the additional molecules of the GGPP metabolite synthesized by the extra molecules of CrtE might inhibit the MEP pathway and/or the studied terpene synthases, similarly to the feedback inhibition caused by the accumulation of IPP [3]. In addition, the increased level of CrtE might negatively influence the hypothetical metabolic pairing of CrtE with the GGPP phosphatase [23], which could somehow act in terpene synthesis.

### 2.5. The Overexpression of the crtE Gene from Synechocystis PCC 6803, but Not Synechococcus PCC 7002, Increases Farnesene Production in Synechococcus PCC 7002

**Figure 6:**
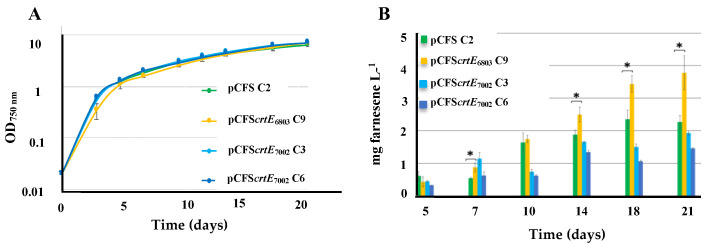
Simultaneous analysis of growth (**A**) and farnesene production (**B**) of *Synechococcus* PCC 7002 strains harboring a pC-derived plasmid strongly expressing either the farnesene synthase gene (pCFS) alone or transcriptionally fused to the *crtE* gene from either *Synechocystis* PCC 6803 (pCFS*crtE*_6803_) or *Synechococcus* PCC 7002 (pCFS*crtE*_7002_). Three clones were studied, as indicated by C3, C6 and C9. Cells were grown under standard photoautotrophic conditions in the presence of a dodecane overlay (20% *v*/*v*) to assay terpene production for 21 days. Error bars represent standard deviation from biological triplicates. They are too small to be visible in panel (**A**), except in one case. The hooks ⊏ indicate a significant difference between the two compared experiments (*t*-test, *p* < 0.05, symbolized by *).

The influence of an extra copy of the *crtE* gene on terpene production in S.7002 was tested as follows. The above-described pCFS*crtE*_6803_ and pCFS*crtE*_7002_ plasmids overexpressing the operonic genes encoding the farnesene synthase and the CrtE enzyme from either S.6803 or S.7002 were introduced by conjugation in S.7002, along with the pCFS control plasmid expressing only the farnesene synthase gene (Figure S6). All three plasmids appeared to replicate stably (Figure S7) without affecting cell growth (Figure 5). As compared to pCFS, pCFS*crtE*6803 and pCFS*crtE*7002, respectively, increased and (slightly) decreased farnesene production in S.7002 (Figure 6). These data suggest that the activity of the CrtE6803 and CrtE7002 enzymes might be different in S.7002.

### 2.6. The Deletion of PHB Synthesis Genes Does Not Increase Terpene Production in Synechocystis PCC 6803.

**Figure 7:**
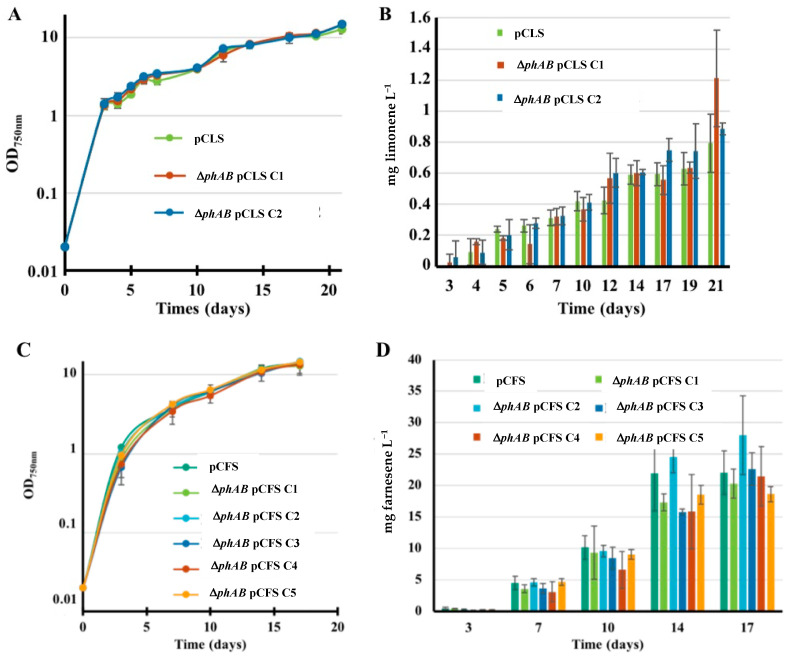
Simultaneous analysis of growth (**A**,**C**) and terpene production (**B**,**D**) of *Synechocystis* PCC 6803 strains WT or Δ*phaAB* mutant harboring a pC-derived plasmid strongly expressing either the limonene synthase gene (pCLS; (**A**,**B**)) or the farnesene synthase gene (pCFS; (**C**,**D**)). Several clones were studied, as indicated by C1–C5. Cells were grown under standard photoautotrophic conditions in the presence of a dodecane overlay (20% *v*/*v*) to assay terpene production for 17 days. Error bars represent standard deviation from biological triplicates. Most of them are too small to be visible in panels (**A**,**C**).

In S.6803, most of the photosynthetically fixed carbon is used for the production of biomass and abundant carbon stores such as glycogen and polyhydroxybutyrates (PHB) [31,32], while only 5% of the carbon is allocated to the MEP pathway for the synthesis of photosynthetic pigments (carotenoids, chlorophyll and quinone) [2,33]. As an attempt to redirect the carbon flux toward the MEP pathway to enhance terpene production in S.6803, we deleted the two adjacent genes *phaAB* encoding the acetoacetyl-CoA thiolase (PhaA) and acetoacetyl-CoA reductase (PhaB) enzymes, which catalyze the first step in PHB synthesis [34,35,36]. Therefore, a Δ*phaAB*::Km^R^ DNA cassette (Table S2) harboring a transcription-terminator less Km^R^ marker in place of most of the *phaA* and *phaB* genes (from codon 93 of *phaA* to codon 141 of *phaB*) was constructed, as follows (Figure S8). The two 300 bp chromosomal DNA regions flanking the *phaAB* DNA region to be deleted were synthesized by TWIST Bioscience as a single DNA segment harboring an *Eco*RV restriction site in its middle where we cloned the Km^R^ marker (a *Hinc*II segment from pUC4K) in the same orientation as the *phaAB* genes that it replaced (Figure S8). The Δ*phaAB*::Km^R^ DNA cassette was verified by PCR and DNA sequencing. It was then transformed to S.6803, where homologous DNA recombination integrated the Km^R^ marker in place of the *phaAB* gene, in all copies of the S.6803 chromosome (Figure S8). Then, the Sm^R^/Sp^R^ plasmids pCFS and pCLS [8,9] were introduced by conjugation in the Δ*phaAB*::Km^R^ mutant (Figure S8 and (Figure S9). In each case, several Sm^R^/Sp^R^/Km^R^ clones were studied. The data showed that the deletion of the *phaAB* genes did not increase the photoproduction of farnesene or limonene in S.6803 (Figure 7).

This approach of deleting PHB synthesis genes to redirect photosynthetically fixed carbons toward terpene production could not be tested in S.7002 because this cyanobacterium does not synthesize PHB [37].

### 2.7. The Deletion of the Carotenoid Synthesis Genes crtR and cruF Decreases the Production of Farnesene in Synechocystis PCC6803

**Figure 8:**
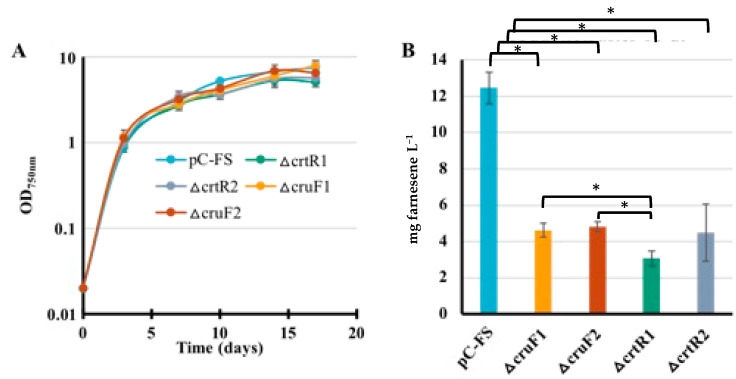
Simultaneous analysis of growth (**A**) and farnesene production (**B**) of *Synechocystis* PCC 6803 WT strain and two clones (1 and 2) of each of the Δ*crtR* and Δ*cruF* mutants harboring the farnesene synthase production pCFS plasmid. Cells were grown under standard photoautotrophic conditions in the presence of a dodecane overlay (20% *v*/*v*) to assay farnesene production (measured at day 10). Error bars represent standard deviation from biological triplicates. They are too small to be visible in panel (**A**). The hooks ⊏ indicate a significant difference between the two compared experiments (*t*-test, *p* < 0.05, symbolized by *).

The production of sesquiterpenes, such as farnesene, competes with the synthesis of photosynthetic pigments (chlorophyll and carotenoids) because they use the same precursor metabolite FPP [38] (Figure 1). To save FPP for a better farnesene production, we decided to decrease the consumption of the FPP-derived metabolite GGPP used for carotenoid synthesis (Figure 1). Therefore, we deleted the genes *crtR* (β-carotene hydroxylase) and *cruF* (γ-carotene hydroxylase) acting in GGPP-consuming synthesis of carotenoids. For *crtR* deletion, a Δ*crtR*::Km^R^ DNA cassette (Table S2) was constructed by replacing the first 82 codons of *crtR* by a transcription-terminator-less Km^R^ marker, as follows. The two 300 bp chromosomal DNA regions flanking the first 82 codons of *crtR* to be deleted were synthesized by TWIST Bioscience as a single DNA segment harboring an *Eco*RV restriction site in its middle, where we cloned the transcription-terminator-less Km^R^ marker (a *Hinc*II segment from the pUC4K plasmid) in the same orientation as *crtR* (Figure S10). The Δ*crtR*::Km^R^ DNA cassette was verified by PCR and DNA sequencing. It was transformed to S.6803, where homologous DNA recombination replaced *crtR* by the Km^R^ marker in all copies of the S.6803 chromosome (Figure S10). The resulting Δ*crtR*::Km^R^ mutant grew as fit as the WT strain under standard light. This finding showed that *crtR* is not essential for the photoautotrophic growth of S.6803, in agreement with previous reports [39,40]. Then, the Sm^R^/Sp^R^ pCFS plasmid was introduced by conjugation in the Δ*crtR*::Km^R^ mutant (Figure S10), and the production of farnesene by two Sm^R^/Sp^R^/Km^R^ clones was analyzed during photoautotrophic growth (Figure 8). The data showed that the deletion of *crtR* does not increase the photoproduction of farnesene in S.6803 (it was decreased), unlike what was expected.

For *cruF* deletion, a Δ*cruF*::Km^R^ DNA cassette (Table S2) was constructed (Figure S12) by replacing an internal part of the *cruF* coding sequence (CS, from codon 100 to codon 271) by the Km^R^ marker. The two 300 bp DNA regions flanking the 171 codons of *cruF* to be deleted were synthesized by TWIST Bioscience as a single DNA segment harboring an *Eco*RV site in its middle, where we cloned the Km^R^ marker in the same orientation as *cruF*. The Δ*cruF*::Km^R^ DNA cassette was verified by PCR and nucleotide sequencing. It was then transformed to S.6803 where homologous DNA recombination replaced the 171 codons of *cruF* by the Km^R^ marker in all copies of the S.6803 chromosome (Figure S12). The resulting Δ*cruF*::Km^R^ mutant grew as fit as the WT strain under standard light showing, for the first time, that *cruF* is not crucial for the photoautotrophic growth of S.6803.

Then, the Sm^R^/Sp^R^ pCFS plasmid was introduced by conjugation in the Δ*cruF*::Km^R^ mutant (Figures S11 and S12), and the farnesene production of two independent Sm^R^/Sp^R^/Km^R^ clones was analyzed (Figure 8). As reported above in the case of *crtR*, the deletion of *cruF* did not increase, but decreased, the photoproduction of farnesene in S.6803 (Figure 8).

Since the strategy of deleting the *crtR* and *cruF* genes did not increase the production of farnesene in S.6803, it was not tested in S.7002.

### 2.8. Deletion of Both the ccmK3 and ccmK4 Genes Encoding Carboxysome Shell Proteins Does Not Alter the Production of Limonene, but Decreases the Production of Farnesene in Synechocystis PCC 6803

In addition to the engineering of genetically modified cyanobacterial organisms (GMOs) for the photosynthetic production of high-value chemicals, it is important to consider strategies to limit accidental release of these GMOs in natural environments. For this purpose, an interesting target is the carbon concentrating mechanism (CCM) that cyanobacteria use to grow in the low CO_2_ concentration of their natural aquatic biotopes. The CCM system uses the carboxysome subcellular compartment, assembled from various Ccm shell proteins, which encapsulates the RubisCO enzyme in a CO_2_-rich environment favoring its carbon-fixing (carboxylase) activity over its detrimental oxygenase activity (for reviews see [4,5]). As a biocontainment strategy, previous workers have deleted carboxysome genes of S.7002 and S.7942 GMO to impose a high-CO_2_ requirement phenotype (HCR) preventing their escape from their cultivation photobioreactors [41,42]. Such a HCR phenotype did not negatively impact L-lactate production in S.7002 [41], but it decreased (about two-fold) farnesene production in S.7942 [42]. Since the HCR containment of GMO has not yet been tested in S.6803, we decided to delete the *ccmK3* and *ccmK4* adjacent genes encoding the CcmK3 and CcmK4 carboxysome shell proteins in S.6803 [43] and S.7942 [44]. These proteins were shown to be required for cell growth at low CO_2_ in S.7942 [44,45], and for optimal photoautotrophic growth in S.6803 [46].

A Δ*ccmK3K4*::Km^R^ DNA cassette (Table S2) was constructed to delete both the *ccmK3* and *ccmK4* colinear genes (from the start of *ccmK3* coding sequence (CS) to the stop codon of *ccmK4*). The 250 bp regions upstream the *ccmK3* CS and downstream the *ccmK4* CS were synthesized by TWIST Bioscience as a single DNA segment harboring *SwaI* and a *Bam*HI restriction sites where we cloned the Km^R^ marker (using the same enzymes) in the same orientation as the *ccmK3* and *ccmK4* CS it replaced (Figure S13). The Δ*ccmK3K4*::Km^R^ grew as healthy as the WT strain in the Na_2_CO_3_-rich MM medium, but was unable to grow under atmospheric (low) CO*_2_* levels (MM_0_ medium), in agreement with previous findings in S.7942 [44,45] and S.6803 [46].

**Figure 9:**
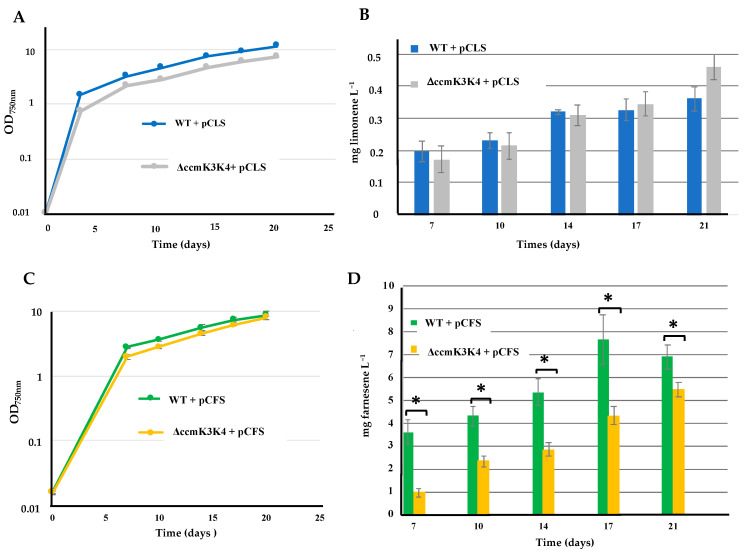
Simultaneous analysis of growth (**A**,**C**) and terpene production (**B**,**D**) of *Synechocystis* PCC 6803 strains WT or Δ*ccmK3K4* mutant harboring a pC-derived plasmid strongly expressing either the limonene synthase gene (pCLS; (**A**,**B**)) or the farnesene synthase gene (pCFS; (**C**,**D**)). Cells were grown under standard photoautotrophic conditions in the presence of a dodecane overlay (20% *v*/*v*) to assay terpene production for 17 or 21 days. Error bars represent standard deviation from biological triplicates. They are too small to be visible in panels (**A**,**C**). The hooks ⊏ indicate a significant difference between the two compared experiments (*t*-test, *p* < 0.05, symbolized by *).

Then, the Sm^R^/Sp^R^ plasmids pCFS and pCLS were introduced by conjugation in the Δ*ccmK3K4*::Km^R^ double mutant, and the production of terpenes in the resulting reporter strains (Figures S13 and S14) was analyzed during photoautotrophic growth in the Na_2_CO_3_-rich MM medium. The data showed that the deletion of *ccmK3K4* did not alter the photoproduction of limonene (monoterpene, C10) in S.6803 (Figure 9), as expected. In contrast, the deletion of *ccmK3K4* decreased the production of farnesene (sesquiterpene, C15). Interestingly, these data showed that the more carbon atoms are used to produce the studied terpene, the more the production is decreased by the Δ*ccmK3K4*::Km^R^ deletion (the production of farnesene (C15) is more important than that of limonene (C10)).

## 3. Materials and Methods

### 3.1. Bacterial Strains and Growth Conditions

*E. coli* strains used for DNA manipulations (TOP10 and NEB10 beta, Table S2) or conjugative transfer to S.6803 and S.7002 (CM404, Ref. [9]) of pC-derived replicative plasmids (Table S2) were grown at 37 °C (TOP10 and NEB10 beta) or 30 °C (CM404) on LB medium containing the selective antibiotics: ampicillin (Amp) 100 μg·mL^−1^, kanamycin (Km) 50 μg·mL^−1^, streptomycin (Sm) 25 μg·mL^−1^ or spectinomycin (Sp) 75 μg·mL^−1^.

S.6803 and S.7002 strains were grown at 30 °C under continuous white light (2500 lux; 31.25 μE·m^−2^·s^−1^) and agitation (140 rpm, Infors rotary shaker) in liquid mineral medium, MM, i.e., BG11 [47] enriched with 3.78 mM Na_2_CO_3_ for S.6803 [9], or A+ supplemented with B12 vitamin (4 μg·L^−1^) for S.7002 [48]. The terpene-producing strains were grown in the presence of the selective antibiotics: Km 50 μg·mL^−1^ for both S.6803 and S.7002, Sm 5 μg·mL^−1^ and Sp 5 μg·mL^−1^ for S.6803, and Sm 50 μg·mL^−1^ and Sp 50 μg·mL^−1^ for S.7002. Growth was monitored by regular measurements of optical density at 750 nm (OD_750_) with a spectrophotometer (Jenway 6700).

### 3.2. Genetic Manipulations and Gene Transfer Techniques

The studied cyanobacterial genes were amplified by polymerase chain reaction (PCR) from cyanobacterial DNA with specific oligonucleotide primers (Table S1) using Hot start Phusion polymerase (ThermoFisher Science France, Illkirch-Graffenstaden, France). PCR products were digested with appropriate restriction enzymes and cloned either in the RSF1010-derived autonomously replicating pC vector for high-level gene expression [9,11], or a commercial *E. coli* plasmid for gene deletion. All DNA constructions were verified by PCR and DNA sequencing (Mix2Seq Kit, Eurofins Genomics France, Nantes, France) using appropriate oligonucleotide primers (Table S1). The pC-derived plasmids (Table S2) were introduced in S.6803 and S.7002 by conjugation [9,11] using a triparental mating where each cyanobacterial strain was coincubated for 72 h with the *E. coli* strains CM404 and a TOP10 (or NEB10 beta) strain propagating a pC-derived plasmid. The DNA deletion cassettes were introduced in S.6803 by transformation [10]. Transformants and conjugants were then plated on MM solidified with 1% Bacto Agar (Difco France, Saint-Ferréol, France), which contained Km 50 μg·mL^−1^ or both Sp 5 μg·mL^−1^ and Sm 5 μg·mL^−1^, respectively, and subsequently incubated at 30 °C under standard light for 7 to 10 days. The presence of the gene deletion cassette, or the terpene-synthase-encoding gene propagated in pC-derived plasmids or a neutral chromosomal site was verified by PCR and DNA sequencing (Mix2Seq Kit, Eurofins Genomics) using appropriate oligonucleotide primers (Table S1).

### 3.3. Terpenes Collection, and Quantification by Gas Chromatography–Mass Spectrometry

S.6803 and S.7002 engineered strains were grown photoautotrophically in the presence of selective antibiotics in 250 mL Erlenmeyer flasks containing 50 mL cell suspensions covered with a nontoxic 20% (*v*/*v*) dodecane overlay (analytical grade, Sigma-Aldrich, St. Louis, MO, USA) to trap terpenes [8,9,11,28,49]. At the specified time intervals, 300 μL of these dodecane overlays was collected, and 1 μL of these samples was injected into a GC–MS apparatus (Trace1300 (GC) + ISQ LT (MS), ThermoScientific) equipped with a TG-5MS column (30 m × 0.25 mm × 0.25 µm) and operated with He carrier gas at 1.0 mL·min^−1^; ionization voltage 70 eV, transfer line temperature 250 °C; ion source temperature 200 °C. Analyses were carried out in the selected ion monitoring mode: *m*/*z* = 50–650, as we previously described, using split modes of 10:1 (limonene) or 5:1 (farnesene). Terpenes were quantified as we previously described [8,9,11].

### 3.4. Statistics

Unless otherwise stated, the data presented represent independent experimental triplicates of the mean and are presented as mean ± standard deviation (SD). The differences between means of the individual groups were analyzed using *t*-test (*p* < 0.05, symbolized by *).

## 4. Conclusions

In this study, we have tested various genetic strategies to attempt to enhance farnesene production in S.6803 and limonene production in S.7002 (Table 1). We report that the overexpression of the genes encoding the key CO_2_-fixing enzymes RubisCO and phosphoribulokinase increase the production of terpenes in S.6803, but not in S.7002. Furthermore, the overexpression of the *crtE* gene (synthesis of terpene precursors) from S.6803, but not S.7002, increases farnesene production in S.7002. In contrast, the overexpression of the *crtE* genes from S.6803 or S.7002 decreases farnesene production in S.6803. Collectively, these results emphasize the physiological differences between S.6803 and S.7002, the two model cyanobacteria that are often used for biotechnology projects [7]. These differences remind us that a model cyanobacterium is essentially a model of itself. Consequently, it is important to study several cyanobacteria in parallel (in the same laboratory) to better understand and compare their common and specific properties, and exploit this knowledge for biotechnological purposes. Here, we also showed that the deletion of the *crtR* and *cruF* genes (carotenoid synthesis) and *phaAB* genes (carbon storage) did not increase the production of farnesene in S.6803. Finally, we have tested a containment strategy for genetically modified organisms (GMOs). We report that the deletion of the *ccmK3K4* genes (carboxysome for CO_2_ fixation) did not impact the production of limonene, but decreased the production of farnesene in S.6803. Collectively, these findings show that the influence on terpene production of the presently tested genetic engineering strategies depends on the nature of the studied cyanobacterial chassis and metabolic enzymes. It is clear from these results and recent data in the literature [20] that one need better understand the metabolism of cyanobacteria to engineer efficient strains for an economically viable photoproduction of terpenes.

## Figures and Tables

**Table 1:** Summary of the terpene producing strains engineered/studied in this study.

Host	Strain Designation	Relevant Features	Terpene and Figure	Yield mg/L
S.6803	ChrFS	Expression of the farnesene synthase gene (FS) cloned in a neutral chromosomal site	Farnesene Figure 2	2.7
	ChrFS + pCrbc6803	ChrFS strain expressing the S.6803 RubisCO genes from the pC plasmid		2.5
	ChrFS + pCrbc7002	ChrFS expressing the S.7002 RubisCO genes from pC		5.2
	ChrFS + pCrbc7942	ChrFS expressing the S.7942 RubisCO genes from pC		2.5
	ChrFS + pCrbcC7425	ChrFS expressing the C.7425 RubisCO genes from pC		4.2
S.6803	ChrFS	Expression of FS	Farnesene Figure 3	2.7
	ChrFS + pCprk7425	ChrFS expressing the C.7425 prk gene from pC		10.2
S.7002	ChrLS	Expression of the limonene synthase gene (LS) from a neutral chromosomal site	Limonene Figure 4	0.8
	ChrLS + pCrbc6803	ChrLS expressing the S.6803 RubisCO genes from pC		0.9
	ChrLS + pCrbc7002	ChrLS expressing the S.7002 RubisCO genes from pC		0.8–0.9
	ChrLS + pCrbc7942	ChrLS expressing the S.7942 RubisCO genes from pC		0.8–0.9
	ChrLS + pCrbcC7425	ChrLS expressing the C.7425 RubisCO genes from pC		0.8–0.9
	ChrLS + pCprk7425	ChrLS expressing the C.7425 prk gene from pC		1.0
S.6803	pCFS	Expression of FS from pC	Farnesene Figure 5	12.0
	pCFScrtE6803	Expression of FS and the S.6803 crtE gene from pC		≤3.0
	pCFScrtE7002	Expression of FS and the S.7002 crtE gene from pC		≤3.0
	pCLS	Expression of LS from pC	Limonene Figure 5	0.55
	pCLScrtE6803	Expression of LS and the S.6803 crtE gene from pC		0.05
	pCLScrtE7002	Expression of LS and S.7002 crtE from pC		0.2
S.7002	pCFS	Expression of FS from pC	Farnesene Figure 6	2.2
	pCFScrtE6803	Expression of LS and S.6803 crtE from pC		3.8
	pCFScrtE7002	Expression of LS and S.7002 crtE from pC		1.8
S.6803	pCLS	Expression of LS from pC	Limonene Figure 7	0.8
	ΔphAB pCLS	Expression of LS from pC and deletion of the phaAB genes		0.9–1.2
	pCFS	Expression of FS from pC		22.0
	ΔphAB pCFS	Expression of FS from pC and deletion of phaAB		20–25
S.6803	pCFS	Expression of FS from pC	Farnesene Figure 8	12.5
	ΔcrtR pCFS	Expression of FS from pC and deletion of the crtR gene		3.7
	ΔcruF pCFS	Expression of FS from pC and deletion of the cruF gene		4.5
S.6803	pCFS	Expression of FS from pC	Farnesene Figure 9	7.0
	ΔccmK3K4 pCFS	Expression of FS from pC and deletion of the ccmK3K4 genes		5.5
	pCLS	Expression of LS from pC	Limonene Figure 9	0.35
	ΔccmK3K4 pCLS	Expression of the LS gene from pC and deletion of ccmK3K4		0.45

## Data Availability

Truly data are contained within the article and Supplementary Materials.

## Supplementary Materials

The supporting information can be downloaded at: https://www.mdpi.com/article/10.3390/ijms25073827/s1. Ref. [50] is cited in Supplementary Materials.
