# Supplementary material for: Impact of Carbon Fixation, Distribution and Storage on the Production of Farnesene and Limonene in Synechocystis PCC 6803 and Synechococcus PCC 7002
Source: Int J Mol Sci. 2024 Mar 29;25(7):3827. doi: 10.3390/ijms25073827 (PMC11012175; doi:10.3390/ijms25073827)
Supplement: Supplementary file 1 [file ijms-25-03827-s001.zip › Figure S1.pptx]

## Slide 1
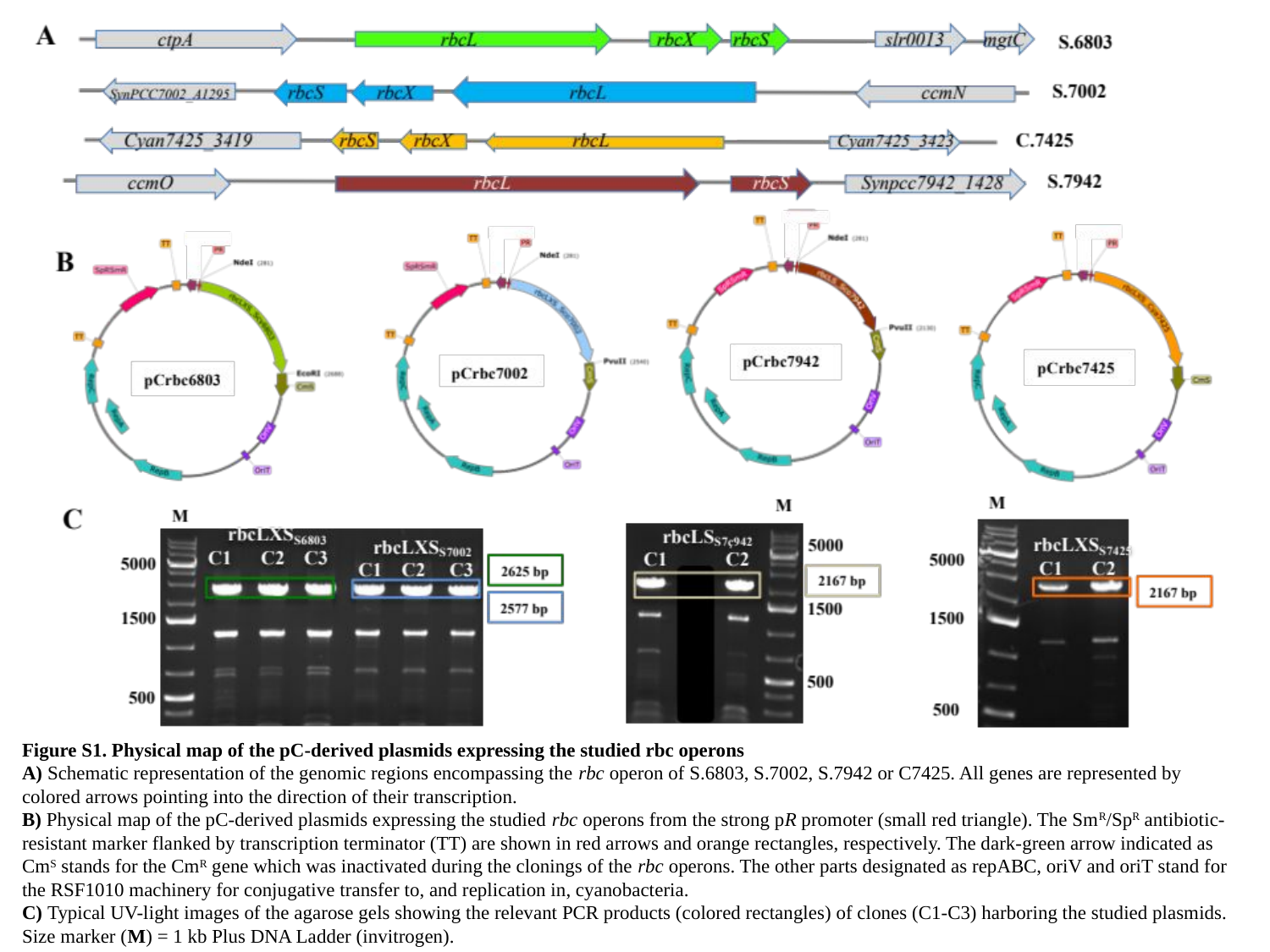

Figure S1. Physical map of the pC-derived plasmids expressing the studied rbc operons
A) Schematic representation of the genomic regions encompassing the rbc operon of S.6803, S.7002, S.7942 or C7425. All genes are represented by colored arrows pointing into the direction of their transcription.
B) Physical map of the pC-derived plasmids expressing the studied rbc operons from the strong pR promoter (small red triangle). The SmR/SpR antibiotic-resistant marker flanked by transcription terminator (TT) are shown in red arrows and orange rectangles, respectively. The dark-green arrow indicated as CmS stands for the CmR gene which was inactivated during the clonings of the rbc operons. The other parts designated as repABC, oriV and oriT stand for the RSF1010 machinery for conjugative transfer to, and replication in, cyanobacteria.
C) Typical UV-light images of the agarose gels showing the relevant PCR products (colored rectangles) of clones (C1-C3) harboring the studied plasmids. Size marker (M) = 1 kb Plus DNA Ladder (invitrogen).
