# Supplementary material for: Impact of Carbon Fixation, Distribution and Storage on the Production of Farnesene and Limonene in Synechocystis PCC 6803 and Synechococcus PCC 7002
Source: Int J Mol Sci. 2024 Mar 29;25(7):3827. doi: 10.3390/ijms25073827 (PMC11012175; doi:10.3390/ijms25073827)
Supplement: Supplementary file 1 [file ijms-25-03827-s001.zip › Figure S10.pptx]

## Slide 1
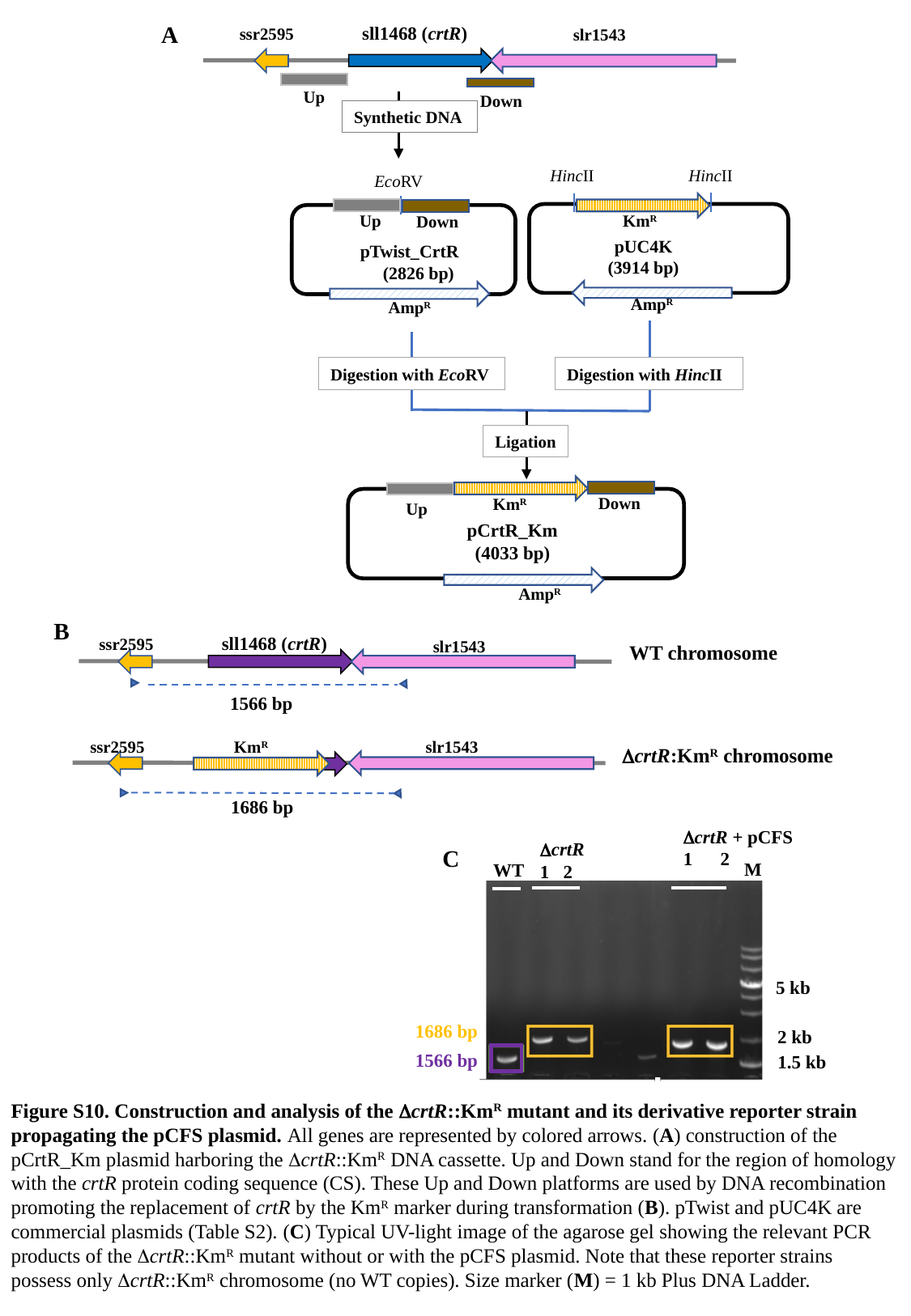

A
ssr2595 sll1468 (crtR)
slr1543
Up
Down
Synthetic DNA
HincII
HincII
EcoRV
Up
KmR
Down
pUC4K
(3914 bp)
pTwist_CrtR
 (2826 bp)
AmpR
AmpR
Digestion with EcoRV
Digestion with HincII
Ligation
Down
KmR
Up
pCrtR_Km
(4033 bp)
AmpR
B
ssr2595 sll1468 (crtR)
slr1543
WT chromosome
1566 bp
ssr2595 KmR
slr1543
DcrtR:KmR chromosome
1686 bp
DcrtR + pCFS
1 2
DcrtR
1 2
C
M
WT
5 kb
1686 bp
2 kb
1566 bp
1.5 kb
Figure S10. Construction and analysis of the DcrtR::KmR mutant and its derivative reporter strain propagating the pCFS plasmid. All genes are represented by colored arrows. (A) construction of the pCrtR_Km plasmid harboring the DcrtR::KmR DNA cassette. Up and Down stand for the region of homology with the crtR protein coding sequence (CS). These Up and Down platforms are used by DNA recombination promoting the replacement of crtR by the KmR marker during transformation (B). pTwist and pUC4K are commercial plasmids (Table S2). (C) Typical UV-light image of the agarose gel showing the relevant PCR products of the DcrtR::KmR mutant without or with the pCFS plasmid. Note that these reporter strains possess only DcrtR::KmR chromosome (no WT copies). Size marker (M) = 1 kb Plus DNA Ladder.
