# Supplementary material for: Impact of Carbon Fixation, Distribution and Storage on the Production of Farnesene and Limonene in Synechocystis PCC 6803 and Synechococcus PCC 7002
Source: Int J Mol Sci. 2024 Mar 29;25(7):3827. doi: 10.3390/ijms25073827 (PMC11012175; doi:10.3390/ijms25073827)
Supplement: Supplementary file 1 [file ijms-25-03827-s001.zip › Figure S11.pptx]

## Slide 1
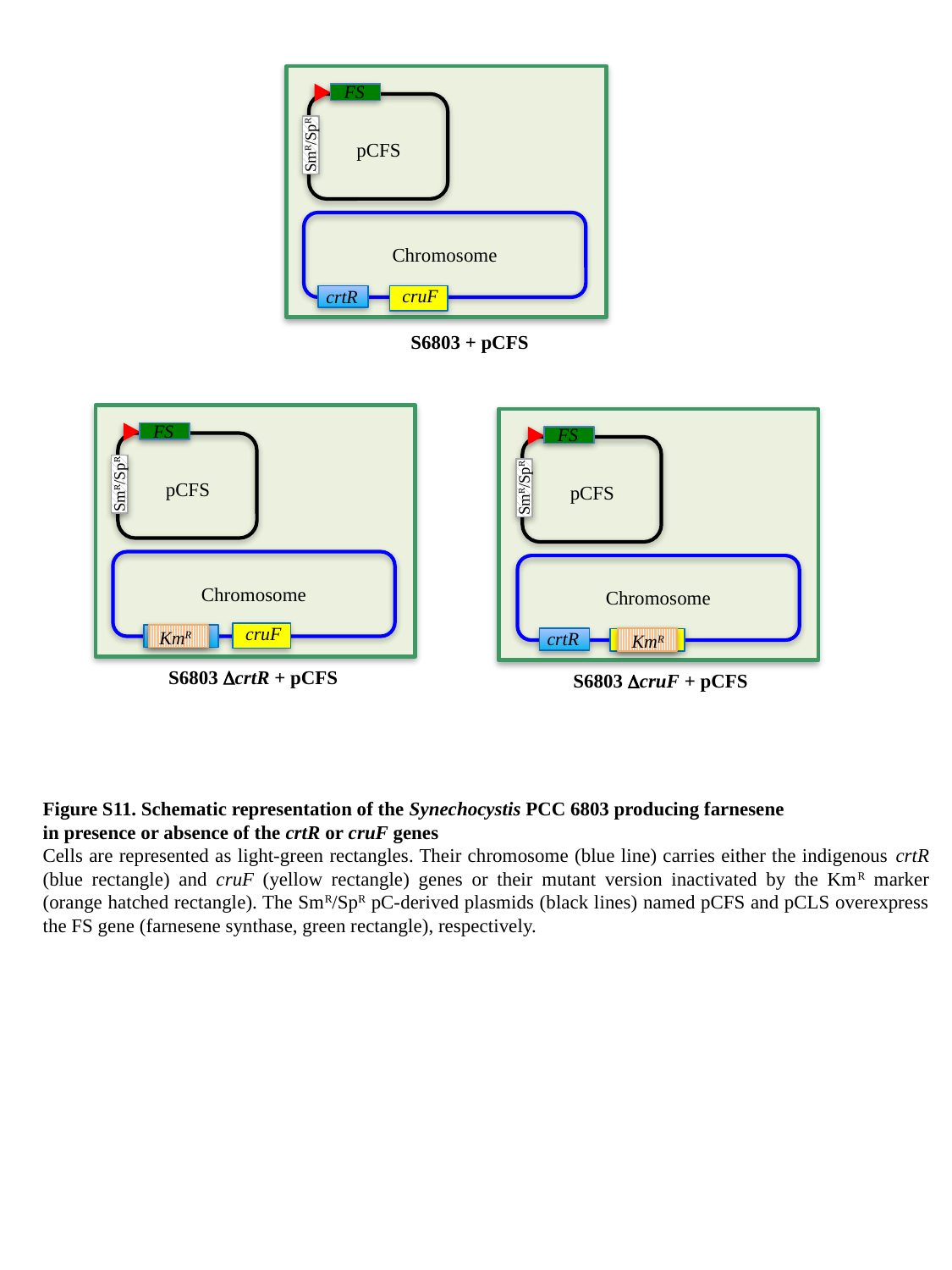

FS
SmR/SpR
pCFS
Chromosome
cruF
crtR
S6803 + pCFS
FS
FS
SmR/SpR
SmR/SpR
pCFS
pCFS
Chromosome
Chromosome
cruF
KmR
crtR
KmR
S6803 DcrtR + pCFS
S6803 DcruF + pCFS
Figure S11. Schematic representation of the Synechocystis PCC 6803 producing farnesene
in presence or absence of the crtR or cruF genes
Cells are represented as light-green rectangles. Their chromosome (blue line) carries either the indigenous crtR (blue rectangle) and cruF (yellow rectangle) genes or their mutant version inactivated by the KmR marker (orange hatched rectangle). The SmR/SpR pC-derived plasmids (black lines) named pCFS and pCLS overexpress the FS gene (farnesene synthase, green rectangle), respectively.
