# Supplementary material for: Impact of Carbon Fixation, Distribution and Storage on the Production of Farnesene and Limonene in Synechocystis PCC 6803 and Synechococcus PCC 7002
Source: Int J Mol Sci. 2024 Mar 29;25(7):3827. doi: 10.3390/ijms25073827 (PMC11012175; doi:10.3390/ijms25073827)
Supplement: Supplementary file 1 [file ijms-25-03827-s001.zip › Figure S12.pptx]

## Slide 1
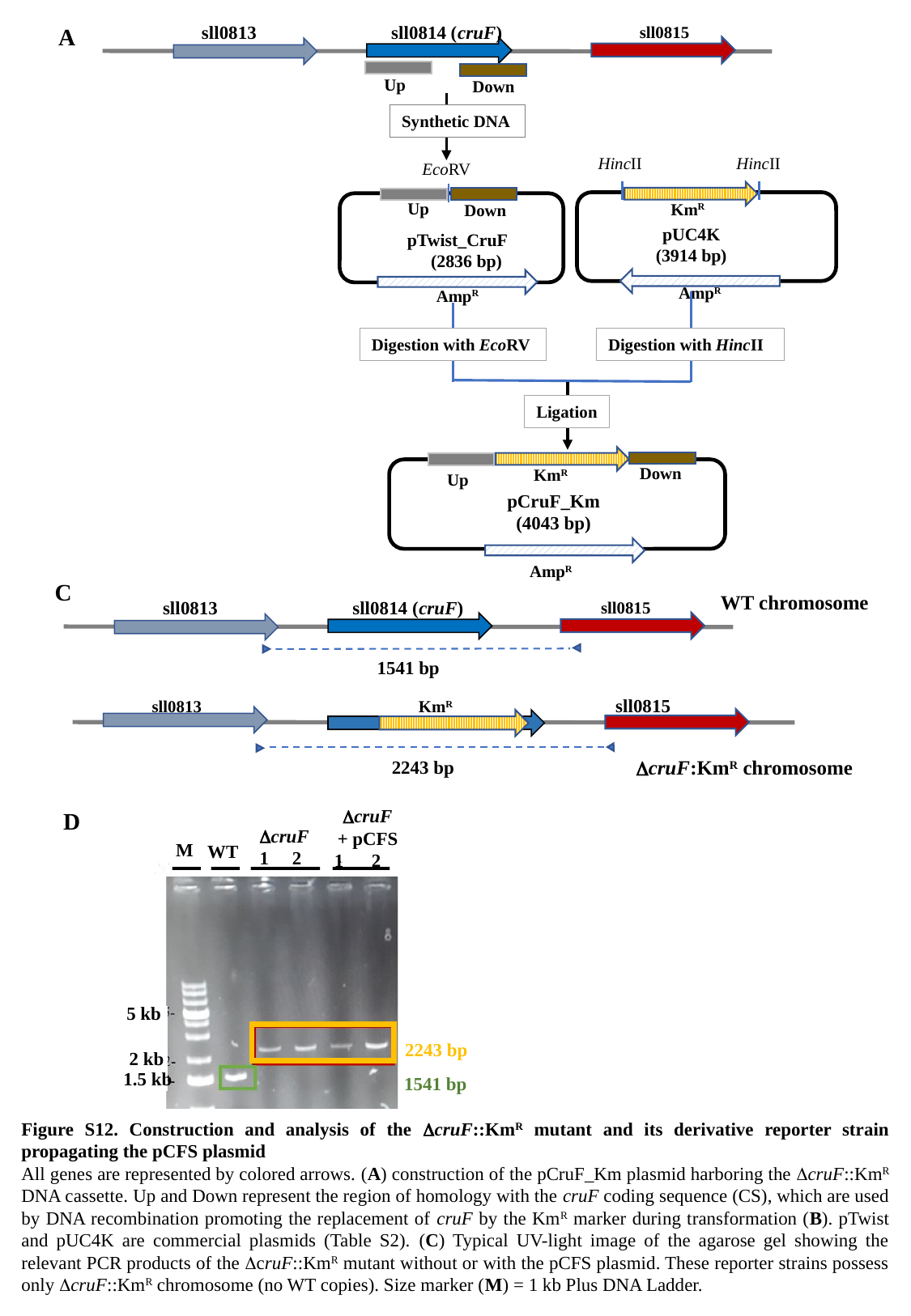

sll0813 sll0814 (cruF)
sll0815
A
Up
Down
Synthetic DNA
HincII
HincII
EcoRV
Up
KmR
Down
pUC4K
(3914 bp)
pTwist_CruF
 (2836 bp)
AmpR
AmpR
Digestion with EcoRV
Digestion with HincII
Ligation
Down
KmR
Up
pCruF_Km
(4043 bp)
AmpR
C
WT chromosome
sll0813 sll0814 (cruF)
sll0815
1541 bp
 sll0813 KmR sll0815
DcruF:KmR chromosome
2243 bp
DcruF
+ pCFS
1 2
D
DcruF
1 2
M
WT
5 kb
2243 bp
2 kb
1.5 kb
1541 bp
Figure S12. Construction and analysis of the DcruF::KmR mutant and its derivative reporter strain propagating the pCFS plasmid
All genes are represented by colored arrows. (A) construction of the pCruF_Km plasmid harboring the DcruF::KmR DNA cassette. Up and Down represent the region of homology with the cruF coding sequence (CS), which are used by DNA recombination promoting the replacement of cruF by the KmR marker during transformation (B). pTwist and pUC4K are commercial plasmids (Table S2). (C) Typical UV-light image of the agarose gel showing the relevant PCR products of the DcruF::KmR mutant without or with the pCFS plasmid. These reporter strains possess only DcruF::KmR chromosome (no WT copies). Size marker (M) = 1 kb Plus DNA Ladder.
