# Supplementary material for: Impact of Carbon Fixation, Distribution and Storage on the Production of Farnesene and Limonene in Synechocystis PCC 6803 and Synechococcus PCC 7002
Source: Int J Mol Sci. 2024 Mar 29;25(7):3827. doi: 10.3390/ijms25073827 (PMC11012175; doi:10.3390/ijms25073827)
Supplement: Supplementary file 1 [file ijms-25-03827-s001.zip › Figure S13.pptx]

## Slide 1
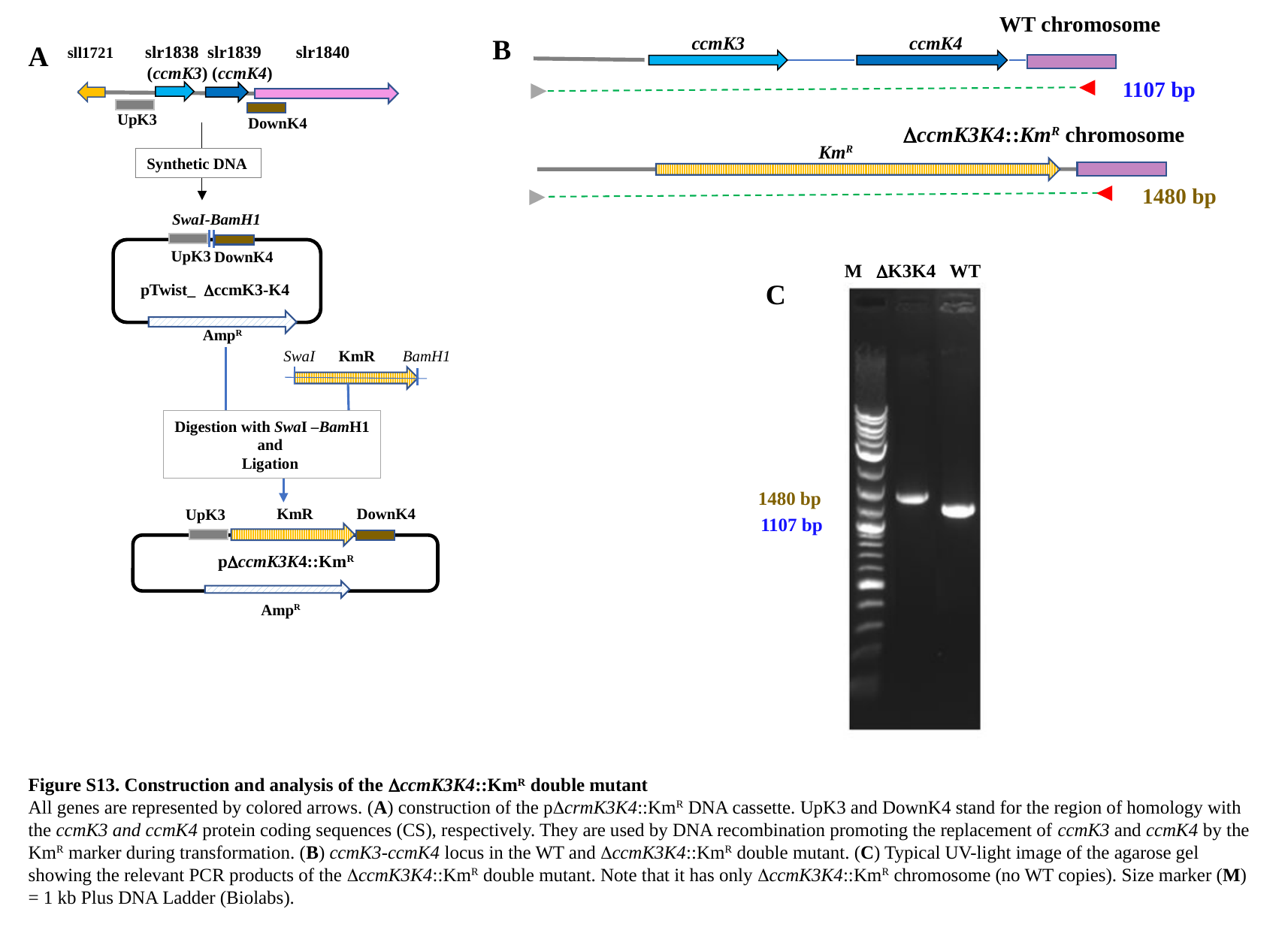

WT chromosome
ccmK3 ccmK4
B
A
 sll1721 slr1838 slr1839 slr1840
 (ccmK3) (ccmK4)
1107 bp
UpK3
DownK4
DccmK3K4::KmR chromosome
KmR
Synthetic DNA
1480 bp
 SwaI-BamH1
UpK3
DownK4
 M DK3K4 WT
C
pTwist_ DccmK3-K4
AmpR
 SwaI KmR BamH1
Digestion with SwaI –BamH1
and
Ligation
1480 bp
1480
 KmR DownK4
UpK3
1107 bp
pDccmK3K4::KmR
1107
AmpR
Figure S13. Construction and analysis of the DccmK3K4::KmR double mutant
All genes are represented by colored arrows. (A) construction of the pDcrmK3K4::KmR DNA cassette. UpK3 and DownK4 stand for the region of homology with the ccmK3 and ccmK4 protein coding sequences (CS), respectively. They are used by DNA recombination promoting the replacement of ccmK3 and ccmK4 by the KmR marker during transformation. (B) ccmK3-ccmK4 locus in the WT and DccmK3K4::KmR double mutant. (C) Typical UV-light image of the agarose gel showing the relevant PCR products of the DccmK3K4::KmR double mutant. Note that it has only DccmK3K4::KmR chromosome (no WT copies). Size marker (M) = 1 kb Plus DNA Ladder (Biolabs).
