# Supplementary material for: Impact of Carbon Fixation, Distribution and Storage on the Production of Farnesene and Limonene in Synechocystis PCC 6803 and Synechococcus PCC 7002
Source: Int J Mol Sci. 2024 Mar 29;25(7):3827. doi: 10.3390/ijms25073827 (PMC11012175; doi:10.3390/ijms25073827)
Supplement: Supplementary file 1 [file ijms-25-03827-s001.zip › Figure S14.pptx]

## Slide 1
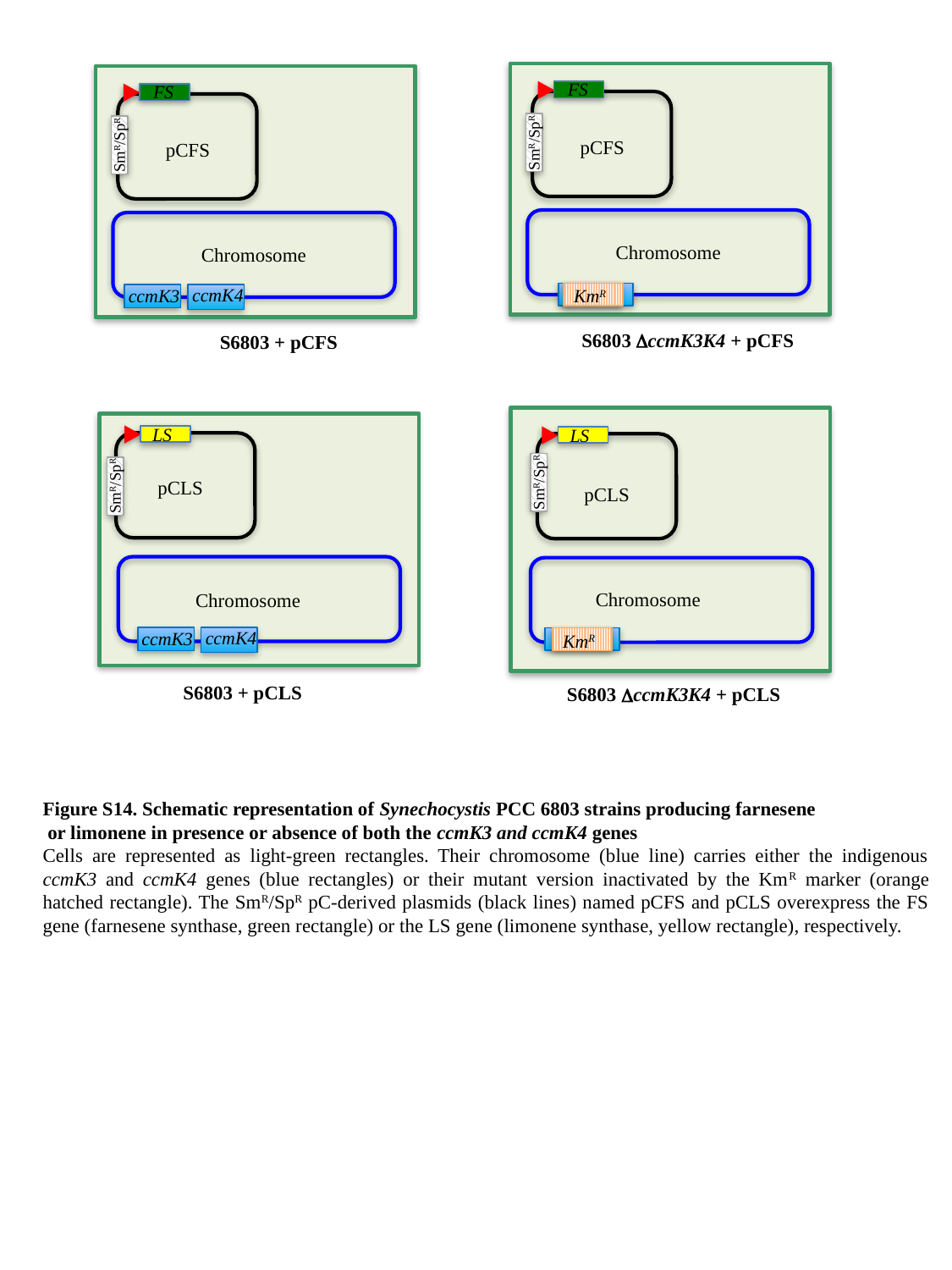

FS
FS
SmR/SpR
SmR/SpR
pCFS
pCFS
Chromosome
Chromosome
ccmK4
ccmK3
KmR
S6803 DccmK3K4 + pCFS
S6803 + pCFS
LS
LS
SmR/SpR
SmR/SpR
pCLS
pCLS
Chromosome
Chromosome
ccmK4
ccmK3
KmR
S6803 + pCLS
S6803 DccmK3K4 + pCLS
Figure S14. Schematic representation of Synechocystis PCC 6803 strains producing farnesene
 or limonene in presence or absence of both the ccmK3 and ccmK4 genes
Cells are represented as light-green rectangles. Their chromosome (blue line) carries either the indigenous ccmK3 and ccmK4 genes (blue rectangles) or their mutant version inactivated by the KmR marker (orange hatched rectangle). The SmR/SpR pC-derived plasmids (black lines) named pCFS and pCLS overexpress the FS gene (farnesene synthase, green rectangle) or the LS gene (limonene synthase, yellow rectangle), respectively.
