# Supplementary material for: Impact of Carbon Fixation, Distribution and Storage on the Production of Farnesene and Limonene in Synechocystis PCC 6803 and Synechococcus PCC 7002
Source: Int J Mol Sci. 2024 Mar 29;25(7):3827. doi: 10.3390/ijms25073827 (PMC11012175; doi:10.3390/ijms25073827)
Supplement: Supplementary file 1 [file ijms-25-03827-s001.zip › Figure S2.pptx]

## Slide 1
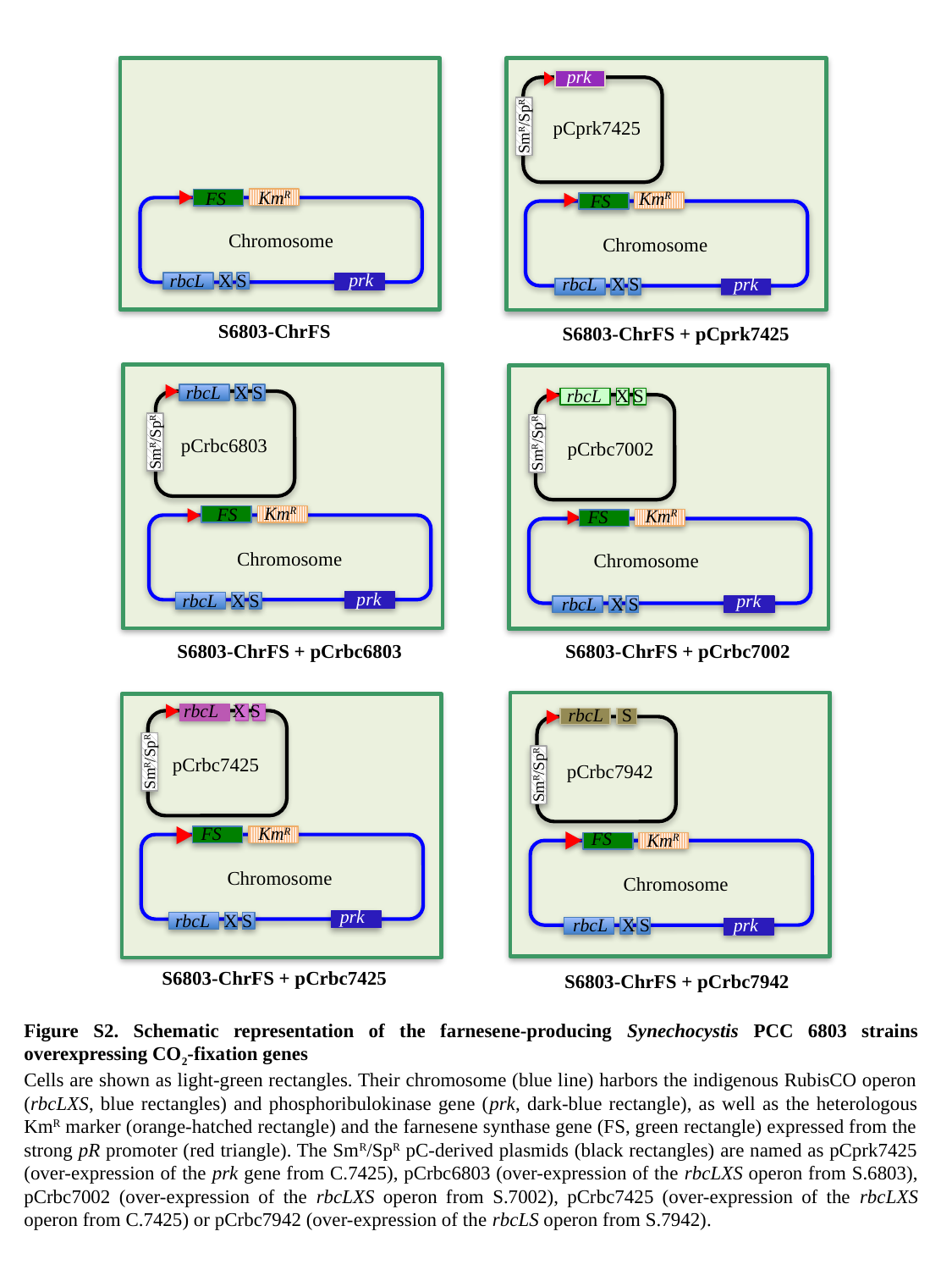

prk
SmR/SpR
pCprk7425
KmR
KmR
FS
FS
Chromosome
Chromosome
 prk
rbcL X S
 prk
rbcL X S
S6803-ChrFS
S6803-ChrFS + pCprk7425
rbcL X S
rbcL X S
prk
SmR/SpR
SmR/SpR
pCrbc6803
pCrbc7002
KmR
 FS
KmR
FS
Chromosome
Chromosome
prk
rbcL X S
prk
rbcL X S
S6803-ChrFS + pCrbc6803
S6803-ChrFS + pCrbc7002
rbcL X S
 rbcL S
SmR/SpR
SmR/SpR
pCrbc7425
pCrbc7942
FS
KmR
FS
KmR
Chromosome
Chromosome
 prk
rbcL X S
rbcL X S
prk
S6803-ChrFS + pCrbc7425
S6803-ChrFS + pCrbc7942
Figure S2. Schematic representation of the farnesene-producing Synechocystis PCC 6803 strains overexpressing CO2-fixation genes
Cells are shown as light-green rectangles. Their chromosome (blue line) harbors the indigenous RubisCO operon (rbcLXS, blue rectangles) and phosphoribulokinase gene (prk, dark-blue rectangle), as well as the heterologous KmR marker (orange-hatched rectangle) and the farnesene synthase gene (FS, green rectangle) expressed from the strong pR promoter (red triangle). The SmR/SpR pC-derived plasmids (black rectangles) are named as pCprk7425 (over-expression of the prk gene from C.7425), pCrbc6803 (over-expression of the rbcLXS operon from S.6803), pCrbc7002 (over-expression of the rbcLXS operon from S.7002), pCrbc7425 (over-expression of the rbcLXS operon from C.7425) or pCrbc7942 (over-expression of the rbcLS operon from S.7942).
