# Supplementary material for: Impact of Carbon Fixation, Distribution and Storage on the Production of Farnesene and Limonene in Synechocystis PCC 6803 and Synechococcus PCC 7002
Source: Int J Mol Sci. 2024 Mar 29;25(7):3827. doi: 10.3390/ijms25073827 (PMC11012175; doi:10.3390/ijms25073827)
Supplement: Supplementary file 1 [file ijms-25-03827-s001.zip › Figure S3.pptx]

## Slide 1
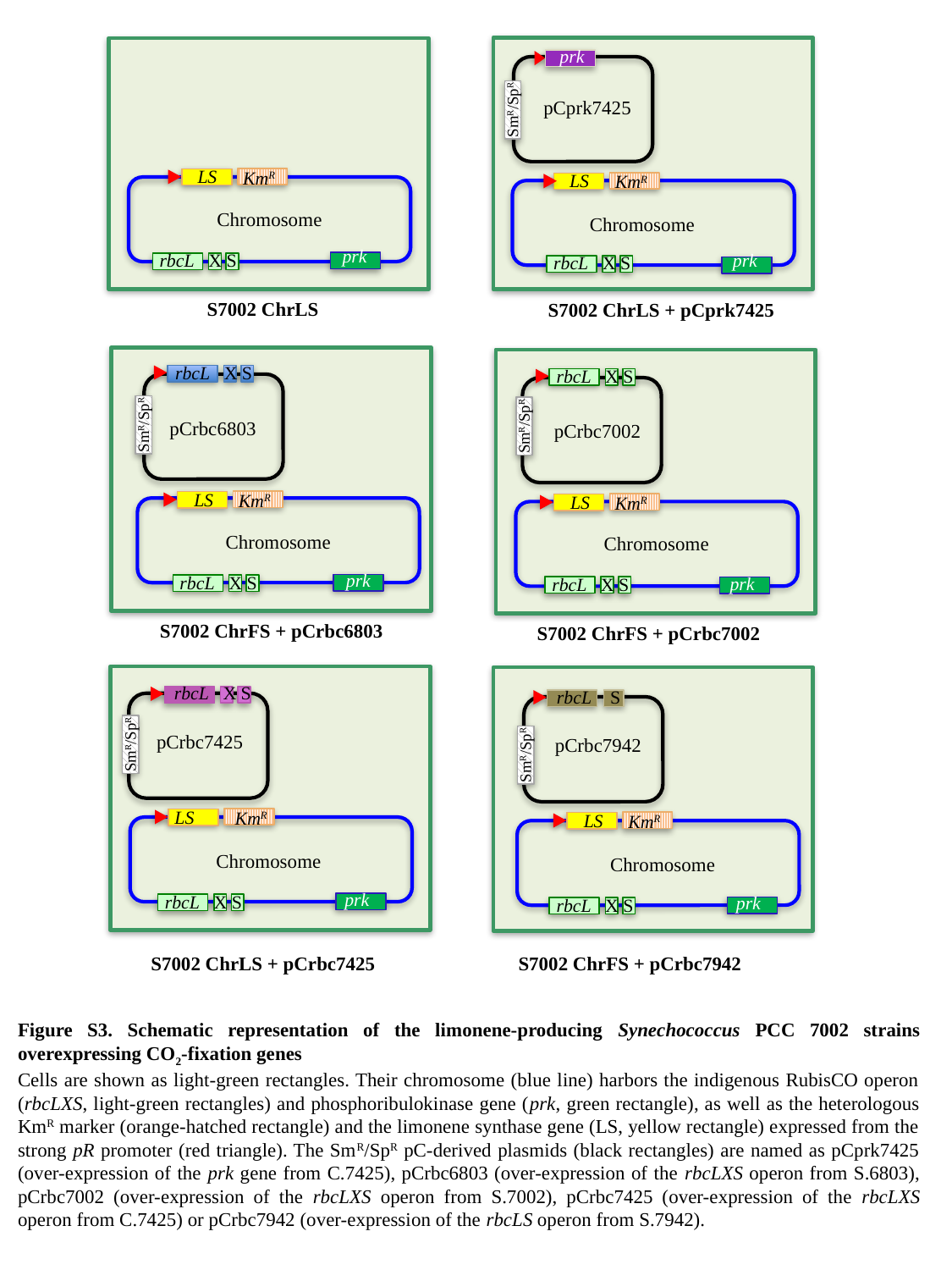

prk
SmR/SpR
pCprk7425
LS
KmR
LS
KmR
Chromosome
Chromosome
prk
 prk
rbcL X S
rbcL X S
S7002 ChrLS
S7002 ChrLS + pCprk7425
rbcL X S
rbcL X S
SmR/SpR
SmR/SpR
pCrbc6803
pCrbc7002
LS
KmR
LS
KmR
Chromosome
Chromosome
prk
rbcL X S
prk
rbcL X S
S7002 ChrFS + pCrbc6803
S7002 ChrFS + pCrbc7002
rbcL X S
 rbcL S
SmR/SpR
SmR/SpR
pCrbc7425
pCrbc7942
LS
KmR
LS
KmR
Chromosome
Chromosome
prk
rbcL X S
prk
rbcL X S
S7002 ChrLS + pCrbc7425
S7002 ChrFS + pCrbc7942
Figure S3. Schematic representation of the limonene-producing Synechococcus PCC 7002 strains overexpressing CO2-fixation genes
Cells are shown as light-green rectangles. Their chromosome (blue line) harbors the indigenous RubisCO operon (rbcLXS, light-green rectangles) and phosphoribulokinase gene (prk, green rectangle), as well as the heterologous KmR marker (orange-hatched rectangle) and the limonene synthase gene (LS, yellow rectangle) expressed from the strong pR promoter (red triangle). The SmR/SpR pC-derived plasmids (black rectangles) are named as pCprk7425 (over-expression of the prk gene from C.7425), pCrbc6803 (over-expression of the rbcLXS operon from S.6803), pCrbc7002 (over-expression of the rbcLXS operon from S.7002), pCrbc7425 (over-expression of the rbcLXS operon from C.7425) or pCrbc7942 (over-expression of the rbcLS operon from S.7942).
