# Supplementary material for: Impact of Carbon Fixation, Distribution and Storage on the Production of Farnesene and Limonene in Synechocystis PCC 6803 and Synechococcus PCC 7002
Source: Int J Mol Sci. 2024 Mar 29;25(7):3827. doi: 10.3390/ijms25073827 (PMC11012175; doi:10.3390/ijms25073827)
Supplement: Supplementary file 1 [file ijms-25-03827-s001.zip › Figure S4.pptx]

## Slide 1
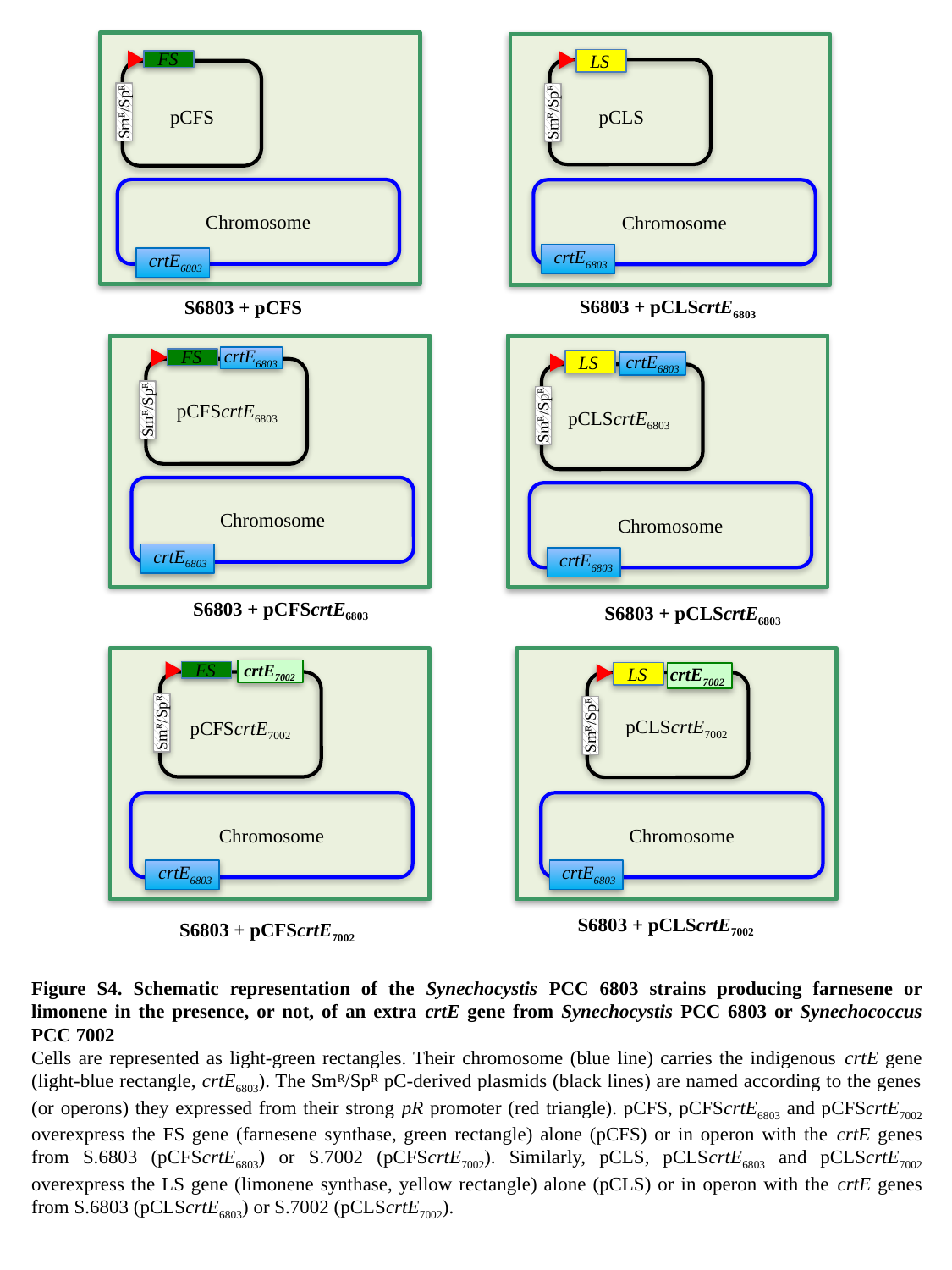

FS
LS
SmR/SpR
SmR/SpR
pCFS
pCLS
Chromosome
Chromosome
crtE6803
crtE6803
S6803 + pCFS
S6803 + pCLScrtE6803
S6803 + pCFS
crtE6803
FS
crtE6803
LS
SmR/SpR
SmR/SpR
pCFScrtE6803
pCLScrtE6803
Chromosome
Chromosome
S6803 DphaAB + pCBS
crtE6803
crtE6803
S6803 + pCFScrtE6803
S6803 + pCLScrtE6803
FS
crtE7002
LS
crtE7002
SmR/SpR
SmR/SpR
pCLScrtE7002
pCFScrtE7002
Chromosome
Chromosome
crtE6803
crtE6803
S6803 + pCLScrtE7002
S6803 + pCFScrtE7002
Figure S4. Schematic representation of the Synechocystis PCC 6803 strains producing farnesene or limonene in the presence, or not, of an extra crtE gene from Synechocystis PCC 6803 or Synechococcus PCC 7002
Cells are represented as light-green rectangles. Their chromosome (blue line) carries the indigenous crtE gene (light-blue rectangle, crtE6803). The SmR/SpR pC-derived plasmids (black lines) are named according to the genes (or operons) they expressed from their strong pR promoter (red triangle). pCFS, pCFScrtE6803 and pCFScrtE7002 overexpress the FS gene (farnesene synthase, green rectangle) alone (pCFS) or in operon with the crtE genes from S.6803 (pCFScrtE6803) or S.7002 (pCFScrtE7002). Similarly, pCLS, pCLScrtE6803 and pCLScrtE7002 overexpress the LS gene (limonene synthase, yellow rectangle) alone (pCLS) or in operon with the crtE genes from S.6803 (pCLScrtE6803) or S.7002 (pCLScrtE7002).
