# Supplementary material for: Impact of Carbon Fixation, Distribution and Storage on the Production of Farnesene and Limonene in Synechocystis PCC 6803 and Synechococcus PCC 7002
Source: Int J Mol Sci. 2024 Mar 29;25(7):3827. doi: 10.3390/ijms25073827 (PMC11012175; doi:10.3390/ijms25073827)
Supplement: Supplementary file 1 [file ijms-25-03827-s001.zip › Figure S6.pptx]

## Slide 1
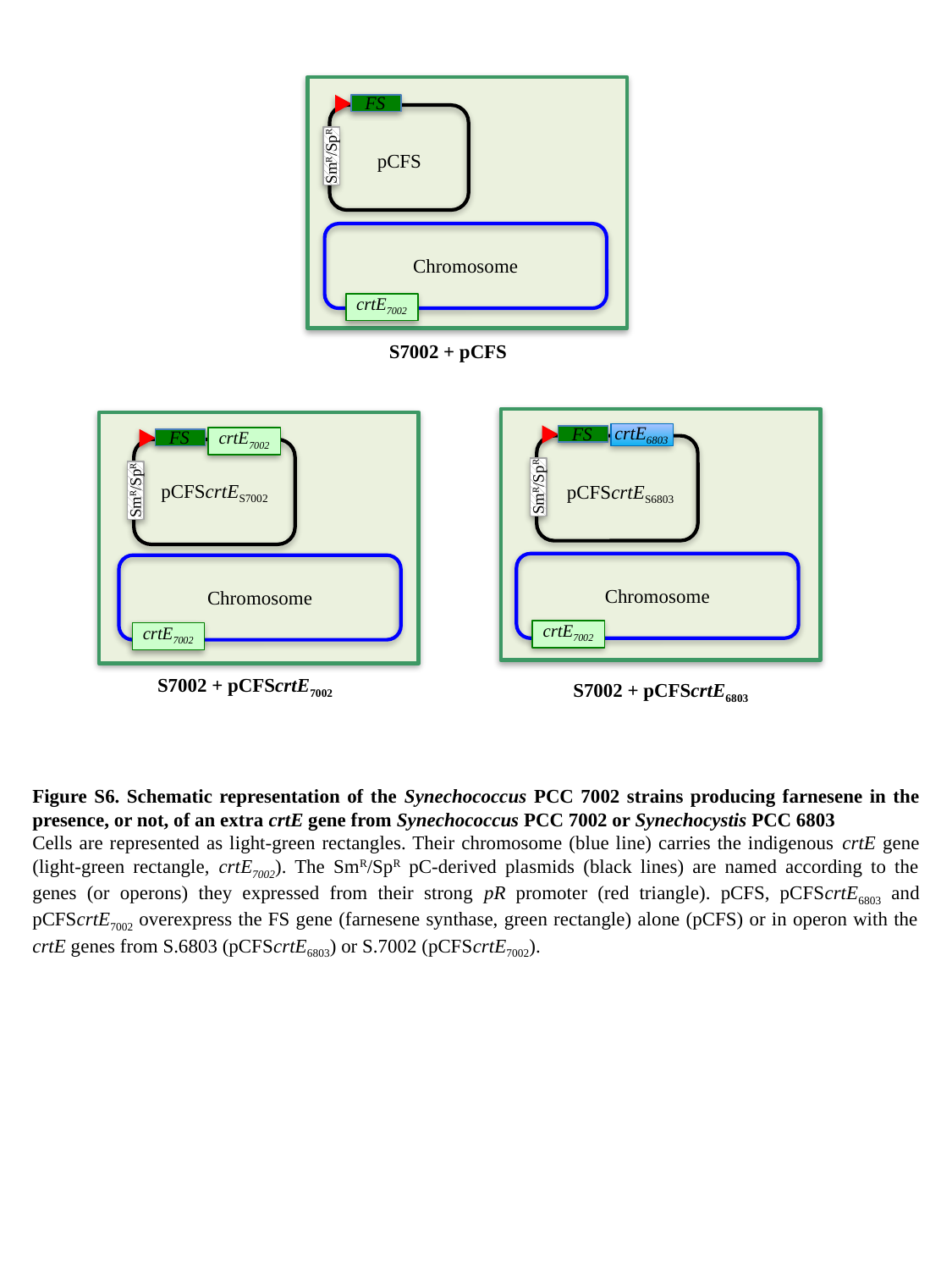

FS
SmR/SpR
pCFS
Chromosome
crtE7002
S7002 + pCFS
crtE6803
FS
FS
crtE7002
SmR/SpR
SmR/SpR
pCFScrtES7002
pCFScrtES6803
Chromosome
Chromosome
crtE7002
crtE7002
S7002 + pCFScrtE7002
S7002 + pCFScrtE6803
Figure S6. Schematic representation of the Synechococcus PCC 7002 strains producing farnesene in the presence, or not, of an extra crtE gene from Synechococcus PCC 7002 or Synechocystis PCC 6803
Cells are represented as light-green rectangles. Their chromosome (blue line) carries the indigenous crtE gene (light-green rectangle, crtE7002). The SmR/SpR pC-derived plasmids (black lines) are named according to the genes (or operons) they expressed from their strong pR promoter (red triangle). pCFS, pCFScrtE6803 and pCFScrtE7002 overexpress the FS gene (farnesene synthase, green rectangle) alone (pCFS) or in operon with the crtE genes from S.6803 (pCFScrtE6803) or S.7002 (pCFScrtE7002).
