# Supplementary material for: Impact of Carbon Fixation, Distribution and Storage on the Production of Farnesene and Limonene in Synechocystis PCC 6803 and Synechococcus PCC 7002
Source: Int J Mol Sci. 2024 Mar 29;25(7):3827. doi: 10.3390/ijms25073827 (PMC11012175; doi:10.3390/ijms25073827)
Supplement: Supplementary file 1 [file ijms-25-03827-s001.zip › Figure S7.pptx]

## Slide 1
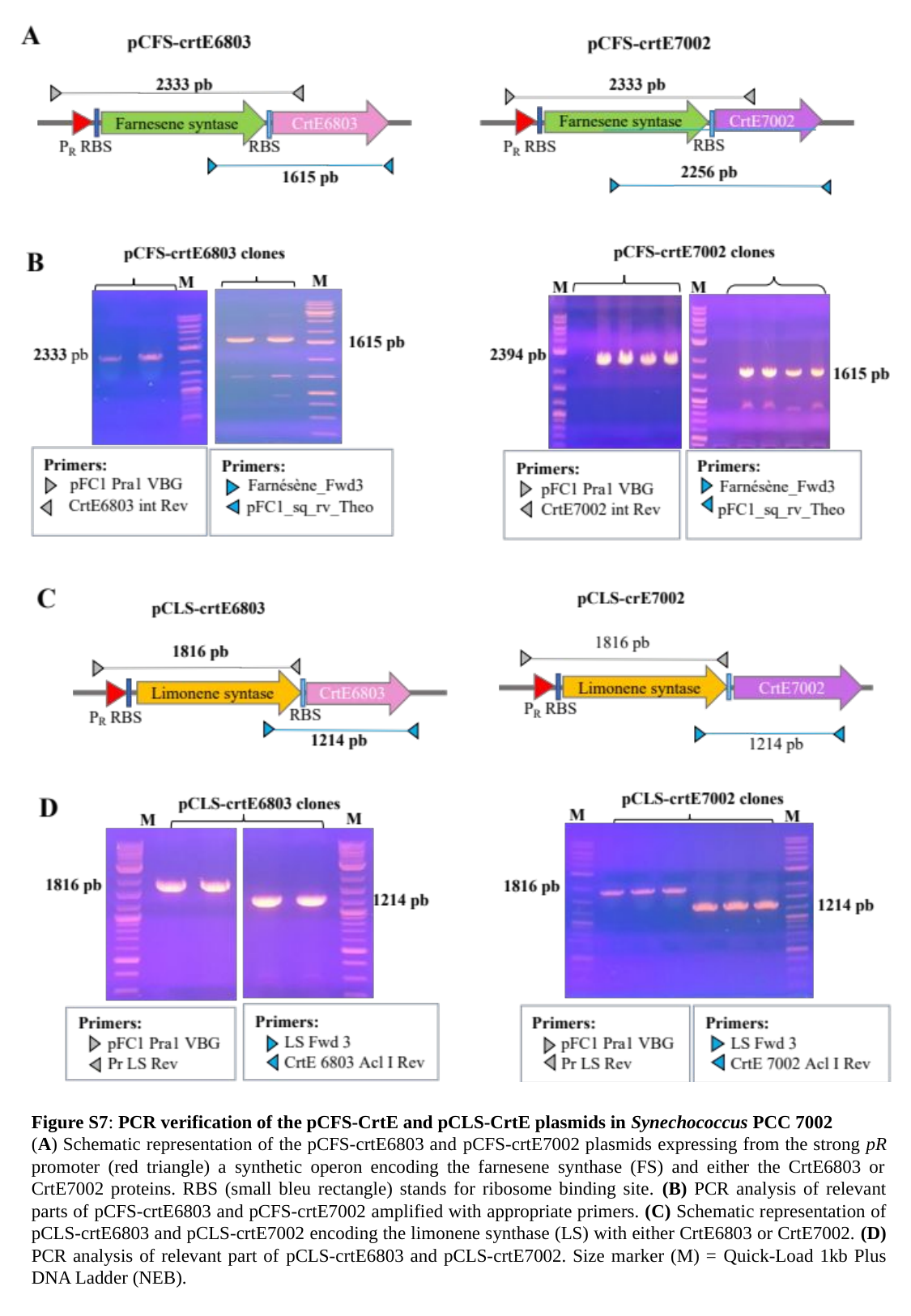

Figure S7: PCR verification of the pCFS-CrtE and pCLS-CrtE plasmids in Synechococcus PCC 7002
(A) Schematic representation of the pCFS-crtE6803 and pCFS-crtE7002 plasmids expressing from the strong pR promoter (red triangle) a synthetic operon encoding the farnesene synthase (FS) and either the CrtE6803 or CrtE7002 proteins. RBS (small bleu rectangle) stands for ribosome binding site. (B) PCR analysis of relevant parts of pCFS-crtE6803 and pCFS-crtE7002 amplified with appropriate primers. (C) Schematic representation of pCLS-crtE6803 and pCLS-crtE7002 encoding the limonene synthase (LS) with either CrtE6803 or CrtE7002. (D) PCR analysis of relevant part of pCLS-crtE6803 and pCLS-crtE7002. Size marker (M) = Quick-Load 1kb Plus DNA Ladder (NEB).
