# Supplementary material for: Impact of Carbon Fixation, Distribution and Storage on the Production of Farnesene and Limonene in Synechocystis PCC 6803 and Synechococcus PCC 7002
Source: Int J Mol Sci. 2024 Mar 29;25(7):3827. doi: 10.3390/ijms25073827 (PMC11012175; doi:10.3390/ijms25073827)
Supplement: Supplementary file 1 [file ijms-25-03827-s001.zip › Figure S8.pptx]

## Slide 1
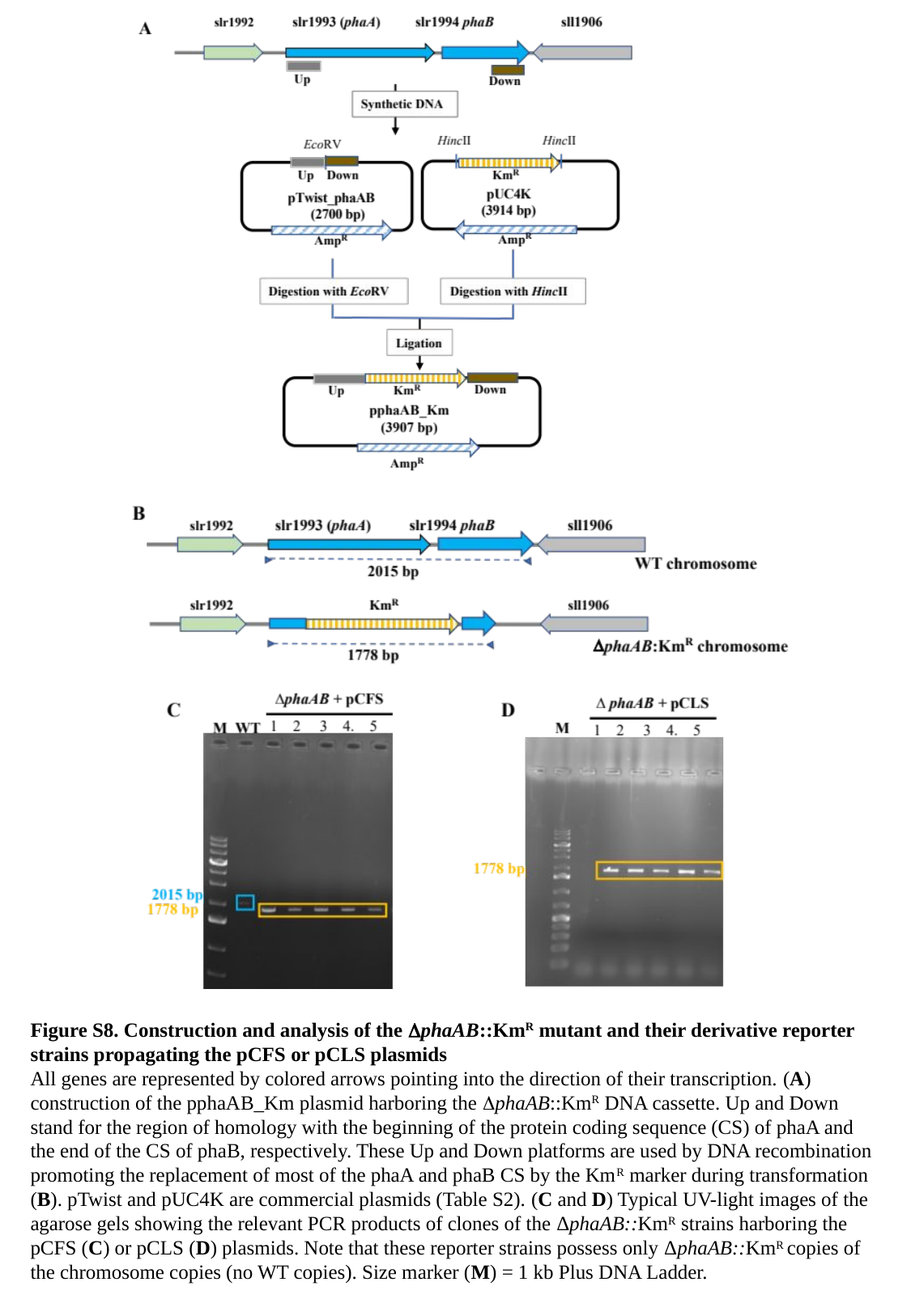

Figure S8. Construction and analysis of the DphaAB::KmR mutant and their derivative reporter strains propagating the pCFS or pCLS plasmids
All genes are represented by colored arrows pointing into the direction of their transcription. (A) construction of the pphaAB_Km plasmid harboring the DphaAB::KmR DNA cassette. Up and Down stand for the region of homology with the beginning of the protein coding sequence (CS) of phaA and the end of the CS of phaB, respectively. These Up and Down platforms are used by DNA recombination promoting the replacement of most of the phaA and phaB CS by the KmR marker during transformation (B). pTwist and pUC4K are commercial plasmids (Table S2). (C and D) Typical UV-light images of the agarose gels showing the relevant PCR products of clones of the ΔphaAB::KmR strains harboring the pCFS (C) or pCLS (D) plasmids. Note that these reporter strains possess only ΔphaAB::KmR copies of the chromosome copies (no WT copies). Size marker (M) = 1 kb Plus DNA Ladder.
