# Supplementary material for: Impact of Carbon Fixation, Distribution and Storage on the Production of Farnesene and Limonene in Synechocystis PCC 6803 and Synechococcus PCC 7002
Source: Int J Mol Sci. 2024 Mar 29;25(7):3827. doi: 10.3390/ijms25073827 (PMC11012175; doi:10.3390/ijms25073827)
Supplement: Supplementary file 1 [file ijms-25-03827-s001.zip › Table S1.pdf]

Table S1 – Characteristics of the oligonucleotides primers used in this study

Name	Sequence (5'-3')	Purpose
**pC and pC derivatives**		
**pC_Fw**	TCATAAATTGCTTTAAGGCG	Forward (Fw) and reverse (Rv) primers for PCR amplification and DNA sequencing of the coding sequences cloned in pC
**pC_Rv**	GTGTAACAAGGGTGAACAC	
**pFC1_Fw**	GGCGACGTGCGTCCTCAAGC	Forward (Fw) and reverse (Rv) primers for PCR amplification and DNA sequencing of the coding sequences cloned in pC
**pFC1_Rv**	GTGTAACAAGGGTGAACAC	
**pFC1_seq_rev_Theo**	CGTTTCAGTTTGCTCATGGA	Forward (Fw) primer for PCR amplification and sequencing of inserts (*prk gene* or rbc operons) cloned in the pC vector.
**Km_HincII_Fw**	GGCGCTGAG**GTCGAC**CTCGTGAAGAAG	Forward (Fw) and reverse (Rv) primers used for Km^r cassette amplification and *Hinc*II restriction sites addition.
**Km_HincII_Rv**	ACCTGCAGGGG**GTCGAC**GGAAAGCCAC	
**Mv13_Fw**	GTAAAACGACGGCCAGT	Forward (Fw) and reverse (Rv) primers using for sequencing plasmids send by Twist Biosciences and verify the insertion of the Km^r cassette in the good direction
Mv13_Rv	CAGGAAACAGCTATGAC	
Km1_Rv	ACGTTTCCCGTTGAATATGGCTC	
**Amplification and cloning of *rbc* operon from various cyanobacteria in the pC vector**		
**rbcLXS_S6803_For(long)**	GTACTA**CATATG**GTACAAGCCAAAGCAGGGTTTAAGGC	Forward (Fw) and reverse (Rv) primers for PCR amplification of rbcLXS operon from S6803 and cloning as a *Nde*I-*Eco*RI DNA fragment in the pC vector
**rbcLXS_S6803_Rev(long)**	TACGTA**GAATTC**TTAGTAACGGCCTTGGTTTTGGTTGGGT	
**rbcLXS_S7002_For(long)**	GTACTA**CATATG**GTTCAGACCAAATCTGCTGGGTTTAAT	Forward (Fw) and reverse (Rv) primers for PCR amplification of rbcLXS operon from S7002 and cloning as a *Nde*I-PvuII DNA fragment in the pCvector
**rbcLXS_S7002_Rev(long)**	TACGTA**CAGCTG**TTAGTAACGGGTTTGGTTGGGCTTGTAAAC	
**rbcLS_S7942_For**	GTACTA**CATATG**CCCAAGACGCAATCT	Forward (Fw) and reverse (Rv) primers for PCR amplification of of rbcLS operon from S7942 and cloning as a *Nde*I-PvuII DNA fragment in the pCvector
**rbcLS_S7942_Rev**	TACGTA**CAGCTG**TTAGTAGCGGCCGGGACG	
**rbcC7425_For**	GTACTA**CTCGAG**ATGTCCTACGCTCAAACA	Forward (Fw) and reverse (Rv) primers for PCR amplification of of rbcLS operon from C7425 and cloning as a XhoI-PvuII DNA fragment in the pCvector
**rbcC7425_Rev**	TACGTA**TCCGGA**TTAGTAGCGATAGCCAC	
**pFC1_RbcS6803(rv_intra)**	TAATTGGTAAATTGCTGTCG	Reverse (Rv) primer for sequencing of the *rbcLXS* operon from S6803 cloned in the pC vector

**pFC1_RbcS7002(rv_intra**	AGCTGCTGACTAATTAGACG	Reverse (Rv) primer for sequencing of the *rbcLXS* operon from S7002 cloned in the pC vector
**pFC1_RbcS7942(rv_intra**	ATGTGCCACACGTGGATAC	Reverse (Rv) primer for sequencing of the *rbcLS* operon from S7942 cloned in the pC vector
**pFC1_RbcC7425(rv_intra)**	TGGTTGTATCTTTGGATATT	Reverse (Rv) primer for sequencing of the *rbcLS* operon from C7425 cloned in the pC vector
**Amplification and cloning in the pC of phosphoribulokinase encoding gene**		
**PRK_C7425_Fwd**	GTACTA**CATATG**ACCTCTAAGCCAGACCGT	Forward (Fw) and reverse (Rv) primers for PCR amplification of prk gene from C7425 and cloning as a *Nde*I-*Eco*RI DNA fragment in the pC vector
**PRK_C7425_Rev**	TACGTA**GAATTC**CTACACCACAGCGGGTTC	
**Construction of the pCFS-CrtE plasmids**		
**CrtE6803_XhoI_Fwd**	AATGGTCA**CTCGAG**AGGAGAACAGCT**ATG**GTTGCCCAACAAACACG	Forward primer for amplification of *crtE* from S.6803 and cloning downstream the stop codon of the FS gene in the pCFS (Table S1). Addition of a *XhoI* site (bold) and a RBS (underlined) before the start codon
**CrtE6803_EcoRI_Rev**	TTATCTCCG**GAATTC**TCAATATTTCTGGCAACAATATATTCGG	Reverse primer for amplification of *crtE* from S.6803 and cloning downstream the FS gene in the pCFS (Table S1). Addition of an *Eco*RI site (bold) after the stop codon (underlined)
**CrtE6803_int_Rev**	TCAAACGCATAGGCTAGCAG	Reverse primer to verify the *FScrtE* operon
**CrtE7002_XhoI_Fwd**	TTTCCAAA**CTCGAG**AGGAGAACAGCT**ATG**GTAGTTGCAGACG	Forward primer for amplification of *crtE* from S.7002 and cloning downstream the stop codon of the FS gene in the pCFS (Table S1). Addition of a *XhoI* site (bold) and a RBS (underlined) before the start codon
**CrtE7002_EcoRI_Rev**	TTGGCACT**GAATTC**TTAGTTTTTACGGTTAACGATGTATTCC	Reverse primer for amplification of *crtE* from S.7002 and cloning downstream the FS gene in the pCFS (Table S1). Addition of an *Eco*RI site (bold) after the stop codon (underlined)
**CrtE7002_int_Rev**	TTCTACGGCAACATCGGTTT	Reverse primer to verify the *FscrtE* operon
**FS_Fwd2**	TTGCTCCGCGCGAGTTACAT	Forward primers to verify the *FscrtE* operon
**FS_Fwd3**	CCAAGGCCGTGATATGTTGA	
**FS_Fwd4**	AGTAATGCGTACCGTACTGG	
**Construction of pCLS-CrtE plasmids**		
**CrtE6803_EcoRI_Fwd**	AATGGT**GAATTC**AGAGGAGAACAGCT**ATG**GTTGCCCAACAAAC	Forward primer for amplification of *crtE* from S.6803 and cloning downstream the stop codon of the LS gene in the pCLS (Table S1).

		Addition of a *Eco*RI site (bold) and a RBS (underlined) before the start codon
**CrtE6803_AclI_Rev**	GTCCATCGTTACGG**AACGTT**TCAATATTTTCTGGCAACAA	Reverse primer for amplification of *crtE* from S.6803 and cloning downstream the FS gene in the pCLS (Table S1). Addition of an *Acl*I site (bold) after the stop codon (underlined)
**CrtE7002_EcoRI_Fwd**	AATGGT**GAATTC**AGAGGAGAACAGCTATGGTAGTTGCAGACGCTCA	Forward primer for amplification of *crtE* from S.6803 and cloning downstream the stop codon of the LS gene in the pCLS (Table S1). Addition of a *Eco*RI site (bold) and a RBS (underlined) before the start codon
**CrtE7002_AclI_Rev**	GTCCCTCGTTACGG**AACGTT**TTAGTTTTTACGGTTAACGATGTATTCC	Reverse primer for amplification of *crtE* from S.7002 and cloning downstream the FS gene in the pCLS (Table S1). Addition of an *Acl*I site (bold) after the stop codon (underlined)
**LS_Fwd3**	TGTCCGATTATAATGCCTCCG	Forward (Fw) and reverse (Rev) primers for amplification of the LS part of *LscrtE* operon
**Pr_LS_Rev**	cctgcaggtc**TTA**AGCAAAGGGCTCAAAC	
**Deletion of *phaAB* in *Synechocystis* PCC 6803**		
**phaAB_Fw**	GCATCCCATTGGAGCGTCG	Forward (Fw) and reverse (Rv) primers used to monitor the segregation of $\Delta phaAB::Km^R$ chromosome in *Synechocystis* PCC 6803
**phaAB_Rv**	CCCAGGGCTTCAGTATTACC	
**Deletion of CrtR in *Synechocystis* PCC 6803**		
**crtR_Fw**	CGTCAATACACCATCTGGCT	Forward (Fw) and reverse (Rv) primers used to monitor the segregation of $\Delta crt::Km^R$ chromosome in *Synechocystis* PCC 6803
**crtR_Rw**	GTGTTGATCTCCTTGATTGG	
**Deletion of *cruF* in *Synechocystis* PCC 6803**		
**cruF_Fw**	ATAGCGAGCTTGTCGTGCCA	Forward (Fw) and reverse (Rv) primers used to monitor the segregation of $\Delta cruF::Km^R$ chromosome in *Synechocystis* PCC 6803
**cruF_Rv**	CCACCTGGGACTCGCCAATA	
**Deletion of *ccmK genes* in *Synechocystis* PCC 6803**		
**CcmK3 FW**	GGCTGGCCACCATAGCG	Forward (Fw) and reverse (Rv) primers used to monitor the segregation of $\Delta ccmK3K4::Km^R$ chromosome in *Synechocystis* PCC 6803
**CcmK4 rev**	GTAACCCACCTTATCTCG	

References:
- Mermet-Bouvier, P.; Chauvat, F.. *Curr. Microbiol.* **1994**, *28*, 145–148, doi:10.1007/BF01571055.

- Chenebault, C. *et al*. *Front. Microbiol.* **2020**, *11*, 586–601, doi:10.3389/fmicb.2020.586601.

- Blanc-Garin, V.; Chenebault, C. et al. .. *Biotechnol. Biofuels* **2022**, *15*, doi:10.1186/s13068-022-02211-0.
