# Supplementary material for: Impact of Carbon Fixation, Distribution and Storage on the Production of Farnesene and Limonene in Synechocystis PCC 6803 and Synechococcus PCC 7002
Source: Int J Mol Sci. 2024 Mar 29;25(7):3827. doi: 10.3390/ijms25073827 (PMC11012175; doi:10.3390/ijms25073827)
Supplement: Supplementary file 1 [file ijms-25-03827-s001.zip › Table S2.pdf]

Table S2 – Characteristics of the bacterial strains and plasmids used in this study

Strain/Plasmid	Relevant features	Reference
Escherichia coli		
**CM404**	*E. coli* strain harboring the self-transmissible plasmid pRK2013 enabling the conjugative transfer of RSF1010-derived pC plasmids into cyanobacteria	(Mermet-Bouvier and Chauvat, 1994)
**TOP10**	*E. coli* strain for cloning	Invitrogen
NEB 5-alpha	*E. coli* strain for cloning	New England Biolabs
**NEB 10-beta**	*E. coli* strain for cloning	New England Biolabs
**Cloning vectors**		
**pTwist**	Amp^R plasmid vector	Twist Biosciences
**pUC4K**	Source of the Km^R marker with no transcription terminator	Pharmacia
**pC**	RSF1010-derived plasmid vector (Sp^R/Sm^R, Cm^R) harboring the strong p_R promoter for constitutive gene expression in *E. coli* and cyanobacteria	(Veaudor et al., 2018)
**Replicative plasmids for strong constitutive expression of terpene synthase genes in cyanobacteria**		
**pCLS**	pC derived plasmid (Sp^R/Sm^R, Cm^S) harboring the *Mentha spicata* 4S-limonene synthase gene (LS) cloned between the *Nde*I and *EcoR*I restriction sites	(Chenebault *et al.*, 2020)
**pCFS**	pC derivative (Sp^R/Sm^R, Cm^S) carrying the *Picea abies* farnesene synthase gene (FS) cloned between *Nde*I and *EcoR*I	(Blanc-Garin *et al.*, 2022)
**Replicative plasmids for strong constitutive expression of terpene synthase encoding genes and *crtE* genes in cyanobacteria**		
**pCLS-CrtE$_{6803}$**	pCLS derived plasmid (Sm^R/Sp^R) harboring the *crtE* gene from S.6803 cloned downstream the LS gene between the *EcoRI* and *AclI* sites	This study
**pCLS-CrtE$_{7002}$**	pCLS derived plasmid (Sm^R/Sp^R) harboring the *crtE* gene from S.7002 cloned downstream the LS gene, between the *EcoRI* and *AclI* sites	This study
**pCFS-CrtE$_{6803}$**	pCFS derived plasmid (Sm^R/Sp^R) harboring the *crtE* gene from S.6803 cloned downstream the FS gene, between the *EcoRI* and *AclI* sites	This study
**pCFS-CrtE$_{7002}$**	pCFS derived plasmid (Sm^R/Sp^R) harboring the *crtE* gene from S.7002 cloned downstream the FS gene, between the *EcoRI* and *AclI* sites	This study
**Plasmids used for deletion of *cruF crtR*, *phaAB*, *ccmK or crtE* in *Synechocystis* PCC 6803**		
**pTwist_CrtR**	pTwist harboring an *Eco*RV restriction site flanked by two platforms of homology for deletion of the *crtR* open reading frame from the ATG start codon to the 245 amino acids of *CrtR* from *Synechocystis* PCC 6803. The 67 last amino acids are conserved to avoid the truncation of *slr1543.*	This study & Twist Biosciences
**pCrtR_Km**	pTwist_CrtR harboring a Km^R cassette cloned in the *EcoRV* site	This study
**pTwist_CruF**	pTwist harboring an *Eco*RV restriction site flanked by two platforms of homology for deletion of the *cruF* open reading frame from the amino acid 100 to the amino acid 271 from *Synechocystis* PCC 6803 to avoid truncation of genes *sll0813 and sll0815*	This study and Twist Biosciences
**pCruF_Km**	pTwist_CruF harboring a Km^R cassette cloned in the *Eco*RV site	This study
**pTwist_phaAB**	pTwist harboring an *Eco*RV restriction site flanked by two platforms of homology for deletion or *phaA* and *phaB* genes	This study and Twist Biosciences

**pphaAB_Km**	pTwist_phaAB harboring a Km^R cassette cloned in the *Eco*RV site	This study
**pTwist_CcmK3K4_Km**	pTwist_CcmK3K4 harboring a Km^R cassette cloned in the *Swa*I et *Bam*HI site	This study and Twist Bioscience
**Replicative plasmids for strong constitutive expression of *rbc* operon or *prk* genes from various cyanobacteria**		
**pC-rbcLXS_Scy6803**	pC derivative plasmid (Sp^R/Sm^R) harboring the ribulose-1,5-bisphosphate carboxylase/oxygenase (RuBisCO) encoding operon (*rbcLXS*) from *Synechocystis* PCC 6803 cloned in between the *Nde*I and *EcoR*I restriction sites	This study
**pC-rbcLS_Sco7942**	pC derivative plasmid (Sp^R/Sm^R) harboring the ribulose-1,5-bisphosphate carboxylase/oxygenase (RuBisCO) encoding operon (*rbcLS*) from *Synechococcus* PCC 7942 cloned in between the *Nde*I and *EcoR*I restriction sites	This study
**pC-rbcLXS_Sco7002**	pC derivative plasmid (Sp^R/Sm^R) harboring the ribulose-1,5-bisphosphate carboxylase/oxygenase (RuBisCO) encoding operon (*rbcLXS*) from *Synechococcus* PCC 7002 cloned in between the *Nde*I and *EcoR*I restriction site	This study
**pC- rbcLXS_Cya7425**	pC derivative plasmid (Sp^R/Sm^R) harboring the ribulose-1,5-bisphosphate carboxylase/oxygenase (RuBisCO) encoding operon (*rbcLXS*) from *Cyanothece* PCC 7425 cloned in between the *Nde*I and *EcoR*I restriction site	This study
**pC-prk_Cya7425**	pC derivative plasmid (Sp^R/Sm^R) harboring the phosphoribulokinase encoding gene (prk) from Cyanothece PCC 7425 cloned in between the *Nde*I and *EcoR*I restriction sites	This study

References:
- Mermet-Bouvier, P.; Chauvat, F.. *Curr. Microbiol.* **1994**, *28*, 145–148, doi:10.1007/BF01571055.

- Chenebault, C. *et al*. *Front. Microbiol.* **2020**, *11*, 586–601, doi:10.3389/fmicb.2020.586601.

- Blanc-Garin, V.; Chenebault, C. et al. .. *Biotechnol. Biofuels* **2022**, *15*, doi:10.1186/s13068-022-02211-0.
